# Modulation of gut microbiota composition and predicted metabolic capacity after nutritional programming with a plant-rich diet in Atlantic salmon (*Salmo salar*): insights across developmental stages

**DOI:** 10.1186/s42523-024-00321-8

**Published:** 2024-07-01

**Authors:** Marwa Mamdouh Tawfik, Marlene Lorgen-Ritchie, Elżbieta Król, Stuart McMillan, Fernando Norambuena, Daniel I. Bolnick, Alex Douglas, Douglas R Tocher, Mónica B. Betancor, Samuel A. M. Martin

**Affiliations:** 1 Scottish Fish Immunology Research Centre, School of Biological Sciences, University of Aberdeen, Aberdeen, AB24 2TZ UK; 2 Hydrobiology Department, Veterinary Research Institute, National Research Centre, Giza, 12622 Egypt; 3 Institute of Aquaculture, University of Stirling, Stirling, FK9 4LA UK; 4 BioMar AS, Havnegata 9, Trondheim, 7010 Norway; 5 Department of Ecology and Evolutionary Biology, University of Connecticut, Storrs, CT 06269-3043 USA; 6 Guangdong Provincial Key Laboratory of Marine Biotechnology, Shantou University, Shantou, 515063 Guangdong China

**Keywords:** Nutritional programming, Intestinal microbiota, Fishmeal, Metabolic programming, Plant-based diet, Vegetable-based diet, Atlantic salmon, First feeding, Microbio, Nutritional history

## Abstract

To promote sustainable aquaculture, the formulation of Atlantic salmon (*Salmo salar*) feeds has changed in recent decades, focusing on replacing standard marine-based ingredients with plant-based alternatives, increasingly demonstrating successful outcomes in terms of fish performance. However, little is known about how these plant-based diets may impact the gut microbiota at first feeding and onwards. Nutritional programming (NP) is one strategy applied for exposing fish to a plant-based (V) diet at an early stage in life to promote full utilisation of plant-based ingredients and prevent potential adverse impacts of exposure to a plant-rich diet later in life. We investigated the impact of NP on gut microbiota by introducing fish to plant ingredients (V fish) during first feeding for a brief period of two weeks (stimulus phase) and compared those to fish fed a marine-based diet (M fish). Results demonstrated that V fish not only maintained growth performance at 16 (intermediate phase) and 22 (challenge phase) weeks post first feeding (wpff) when compared to M fish but also modulated gut microbiota. PERMANOVA general effects revealed gut microbiota dissimilarity by fish group (V vs. M fish) and phases (stimulus vs. intermediate vs. challenge). However, no interaction effect of both groups and phases was demonstrated, suggesting a sustained impact of V diet (nutritional history) on fish across time points/phases. Moreover, the V diet exerted a significant cumulative modulatory effect on the Atlantic salmon gut microbiota at 16 wpff that was not demonstrated at two wpff, although both fish groups were fed the M diet at 16 wpff. The nutritional history/dietary regime is the main NP influencing factor, whereas environmental and host factors significantly impacted microbiota composition in M fish. Microbial metabolic reactions of amino acid metabolism were higher in M fish when compared to V fish at two wpff suggesting microbiota played a role in digesting the essential amino acids of M feed. The excessive mucin O-degradation revealed in V fish at two wpff was mitigated in later life stages after NP, suggesting physiological adaptability and tolerance to V diet. Future studies are required to explore more fully how the microbiota functionally contributes to the NP.

**Supplementary Information:**

The online version contains supplementary material available at 10.1186/s42523-024-00321-8.

## Introduction

The use of plant-based proteins and oils as substitutes for marine-based ingredients in fish feed is increasing due to a finite supply of fishmeal (FM) and fish oil (FO) [1–3] that is causing unsustainable pressure on ocean ecosystems [4]. However, these substitutions can negatively impact fish performance as a plant-based diet is considered to have less than optimum nutritional quality [5]. Studies on plant-based diets revealed the presence of anti-nutritional factors, unbalanced amino acid profiles [6–9] and low levels of long-chain polyunsaturated fatty acids (LC-PUFA) particularly omega-3 fatty acids (including EPA and DHA) [10, 11]. Thus, strategies such as supplementing with essential fatty acids, balancing amino acid profiles, and reducing anti-nutritional factors in plant-based diet have been implemented [12–18]. Nutritional programming (NP) has shown promise as an approach to promote more efficient usage of plant materials in non-herbivorous mammals [5, 19] and recently in fish [13, 19–26]. NP studies utilising marine vs. plant-based diets and their effects on fish performance and physiology have been carried out in Atlantic salmon (*Salmo salar*) [20] and rainbow trout (*Oncorhynchus mykiss*) [21]. In Atlantic salmon, the latter group improved their ability to utilise the diet, resulting in a 24% higher growth rate and 23% enhanced feed efficiency [20]. Similarly, plant-programmed rainbow trout exhibited a 42% higher growth rate, 18% higher feed efficiency, and a 30% higher feed intake [21].

Microbiota are essential for host development, immunity, energy and mucosal homeostasis, and metabolism [27–32]. The gut microbiota responds to dietary and environmental changes and plays a crucial role in gut functioning [33–35]. Although studies aim to comprehend the interaction between microbiota and their host in response to dietary nutrients [36, 37], research on the impact of plant-based feed ingredients on early-life stages of Atlantic salmon microbiota is limited [6, 38, 39]. While previous studies have mainly focused on soybean products, fewer studies have been directed towards plant protein concentrates or plant-sourced mixtures [40–43]. Mixing dietary plant ingredients produced a less inflammatory response and better plant-based diet usage [5, 44].

Studies have explored the relationship between NP with plant-based ingredients and the gut bacterial microbiota (named microbiota across this study unless otherwise stated) in fish, but showed no significant links using 16 S rRNA sequencing [45–49]. On the other hand, fungal microbiota of the gut was modulated on a short- and long-term basis and that accompanied the history of 5-day hyperglucidic hypoproteic nutritional stimulus during first exogenous feeding in rainbow trout using DGGE [50]. Rainbow trout and brown trout (*Salmo trutta*) showed no sustained changes in their gut microbiota when fed different levels of plant-derived proteins at first feeding [47, 48] while in a study on zebrafish (*Danio rerio*) larvae, pre-adult fish fed with plant-based diets had a higher weight gain than those fed with FM diets but without significant microbiota changes contributing to NP effects [49]. Although plant-based NP studies have identified differences in growth and utilisation, gut microbial changes associated with this NP have not been detectable to date in various fish species using 16 S rRNA sequencing [46, 49].

In this study, we investigated the impact of NP with a plant-based (V) diet at first feeding in Atlantic salmon on gut microbial composition, diversity, and potential microbial community functions, compared to fish fed a traditional marine-based (M) diet. We fed the fish for a brief period of two weeks at first feeding with either a V or M diet (stimulus phase), then with a M diet for 14 weeks (intermediate phase) before challenging with a V diet (challenge phase) and sampled both gut and its contents (mucosa and digesta). We used 16 S rRNA gene amplicon sequencing to monitor the composition of the gut microbiota. We found microbiota dissimilarity between the fish initially fed marine (M fish) and plant (V fish) diets, which may be involved in maintaining the specific growth rate after plant-based NP (16 wpff) as we recently demonstrated [51].

## Materials and methods

### Feeding trial, diets, and sampling

Before the work was conducted, the Animal Welfare and Ethical Review Board, University of Stirling [AWERB (18 19) 045 New ASPA] ethically reviewed all experiments. The Atlantic salmon eggs were obtained from Mowi, Norway. The feeding trial was conducted in 2019 in the fry stage during the first exogenous feeding at the Institute of Aquaculture (University of Stirling) in a temperate recirculating aquaculture system (RAS) with M and V diets. The feeding lasted for 22 weeks and consisted of three phases: (1) stimulus, (2) intermediate and (3) challenge. Before sampling, the fish was humanely sacrificed by water bath immersion in MS222 anaesthetic at a concentration sufficient for overdose.

**Fig. 1:**
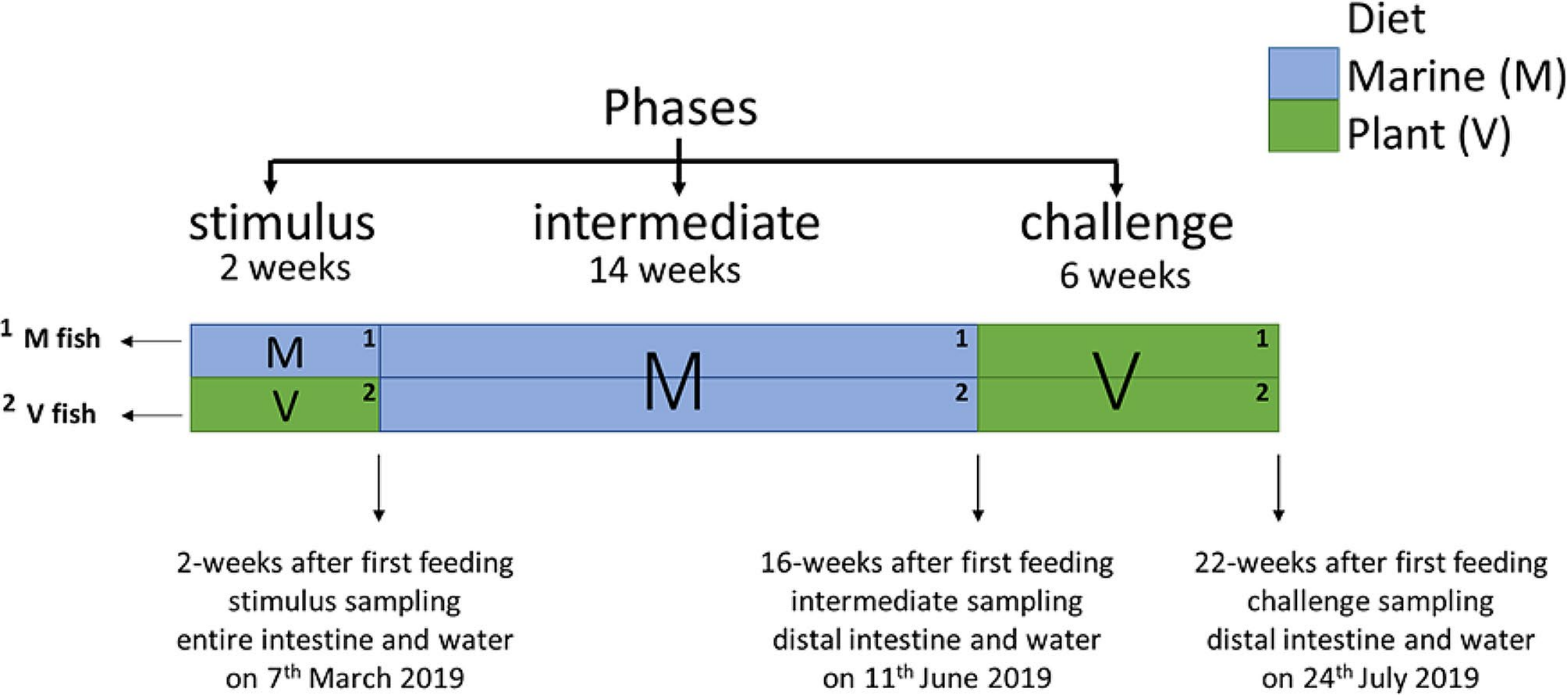
Details of dietary manipulations performed on Atlantic salmon with three sampling points for microbiota samples. The gut (*n* = 108) and water (*n* = 18) were collected at sampling points and feed (*n* = 6) at the start of the feeding trial. Experimental groups/dietary regimes are M fish/water and V fish/water for intestinal and water samples

BioMar AS at the BioMar Tech Centre (Brande, Denmark) manufactured all diets. The M diet was rich in FM and FO, whilst the V diet was rich in plant protein concentrates and rapeseed oil (low FM/FO). The diets were adapted to reflect the size of the fish (Table S1, McMillan et al. [51]). Fish were dietary stimulated (stimulus phase; two weeks) at first feeding with the V diet vs. M diet and later challenged (challenge phase; six weeks) with a similar V diet after an intermediate period on the M diet (intermediate phase; 14 weeks). The fish/intestine were sampled (*n* = 18/treatment/phase, *n* = 108 in total) and tank water (*n* = 3/treatment/phase, *n* = 18 in total) at the end of each feeding phase, producing three sampling points: stimulus (two wpff), intermediate (16 wpff), and challenge (22 wpff) (Fig. 1). Feed samples were collected (*n* = 3 for each of the M and V diets) before the start of the feeding trial.

The whole fish from stimulus sampling and distal intestine from intermediate and challenge sampling points were preserved in 1.5 ml RNAlater™ (Ambion Inc., United States) in 2 ml graduated safelock microcentrifuge tubes (Thermo Scientific, UK). Samples were stored at 4 °C for 24 h, followed by longer-term storage at − 80 °C. The gut along with any associated digesta (100–150 mg at intermediate and challenge sampling points) was excised aseptically from the fish whereas due to size constraints the whole intestine (≈ 20 mg) from whole fish preserved in RNAlater™ at stimulus sampling was used for DNA extraction (Fig. S1). Tank water (50–100 ml) was collected at each sampling point and stored at − 20 °C within 12 h of sampling before filtering through 0.2 µM Whatman Cyclopore polycarbonate membrane filters (Sigma-Aldrich; WHA70634702) using a vacuum pump. Filters and feed samples were stored at − 80 °C prior to downstream processing and DNA extraction.

### DNA extraction

The intestinal samples were thawed on ice and removed excess RNAlater by gently squeezing the tissue, which was then transferred to a 2 ml Eppendorf tube for DNA extraction. DNA was then extracted using the QIAamp® DNA Stool Mini Kit (Qiagen). Some adjustments, as described by Dehler et al. [38], were made to the manufacturers’ protocol to aid in the lysis of tough-walled Gram-positive bacteria. Intestinal samples were heated to 95 °C in Inhibit Ex buffer and mechanically lysed using two 3 mm tungsten carbide beads (Qiagen) and a TissueLyser for 4 min (30 Hz frequency). DNA was eluted in a final volume of 30 µl. DNA was extracted from water filters and diets (200 mg of pellets) using the same protocol. DNA quantity was determined, and purity was assessed by NanoDrop spectrometry (Thermo Scientific). Extracted DNA samples were kept at -20° C until further processing.

### 16 S rRNA PCR amplification and sequencing

The V3-V4 hypervariable regions of the 16 S rRNA gene were targeted with the 341 F/785R primer pair [52] for PCR amplification. Illumina adapter overhang sequences were added to the 5′ ends of each primer. 5′ TCG TCG GCA GCG TCA GAT GTG TAT AAG AGA CAG **CCT ACG GGN GGG CWG CAG** was the forward primer (341 F) sequence, and 5′ TCT CGT GGG CTC GGA GAT GTG TAT AAG AGA CAG **GAC TAC HVG GGT ATC TAA TCC** was the reverse primer (785R) sequence with the bold underlined sequence being the locus-specific V3-V4 primers. A previously described protocol [53] was followed with some modifications described here. Each PCR reaction was carried out in a 10 µl volume [1 µl of DNA, 2 µl of forward and 2 µl of reverse primers (1 µM stock, Sigma), and 5 µl of 2x KAPA HiFi HotStart ReadyMix (KAPA Biosystems Ltd., UK)]. Thermal cycling conditions were as follows: an initial denaturation at 95 °C for 3 min, followed by 26 cycles of 30 s at 98 °C, 30 s at 57 °C, and 30 s at 72 °C after which a final extension (at 72 °C for 5 min) was applied. Samples were randomised to prevent batch effects and amplified in triplicate 96-well plates. Triplicate reactions were pooled following amplification. Diet samples were diluted to 200 ng µl^–1^ before PCR amplification. A subset of amplified products was run on an Agilent 2200 TapeStation (Agilent Technologies, Italy) to confirm that the integrity and concentration of DNA suitability for sequencing. Positive (16 S rRNA gene reference) and DNA-negative controls were sequenced alongside each plate (two plates in triplicates) in addition to a blank membrane filter (a negative control for water samples). PCR products were sequenced at CGEBM, University of Aberdeen. Paired-end (2 × 300 bps) sequencing was performed using the Illumina MiSeq platform (see [53] for further details).

### Bioinformatic and statistical analyses

Illumina adapters, along with external primers (first 17 bps of forward reads and first 21 bps of reverse reads), were removed from the pair-ended reads using TrimGalore! (v0.6.4 [54]). Trimming V3-V4 primers from within the sequences was performed by Cutadapt v4.0 [55] for more stringent cleaning. The low-quality reads (Phred quality score < Q30) were discarded, and the forward and reverse reads were truncated at 250 and 220 bps, respectively (Fig. S2). Following the adaptor and primer removal, and quality filtering, reads were analysed using the DADA2 pipeline [56] to identify the microbial community as Amplicon Sequence Variants (ASVs). To assign taxonomic classification, reads were aligned against the SILVA v138 database [57]. Only the kingdom Bacteria was selected for further analysis (Archaea and Eukarya were excluded). Known contaminants (mitochondria, cyanobacteria and chloroplasts) were pruned out. Sequence contaminants were removed by decontam v1.14 [58] while the undetected contaminant Methylobacterium-Methylorubrum [59] was discarded manually. Singletons that arose during merging forward and reverse reads were also manually filtered out [60]. Samples with less than 1000 reads were further excluded resulting in 129 samples in total for downstream analysis (*n* = 105 gut, *n* = 6 feed, and *n* = 18 water samples). For the alpha diversity measure, reads were normalised by scaling with ranked subsampling (SRS [61]), using microeco (v0.20.0 [62]), at the minimum of read counts. Alpha diversity was estimated as the Shannon-Weiner index using the estimate_richness function (phyloseq v1.38 [63]). Beta diversity analysis to understand differences in microbiota composition and diversity between M and V fish at different sampling times was performed on a natural log-transformed dissimilarity matrix of Bray-Curtis distances (BC [64]). Additionally, to understand differences between intestine, feed and water, a robust Aitchison distance metric was performed on clr-transformed samples, taking into account the phylogenetic trees when calculating distances. Principal coordinate analysis (PCoA) was performed on the BC dissimilarity matrix for visualising similarity/dissimilarity between intestinal samples. Taxonomic microbial composition was normalised by transforming abundances into relative abundances (RA). Unique and overlapping ASVs between gut and water or feed were detected at a minimum of 0.05% RA. Metabolic reaction analysis of intestinal microbiota was performed in Python 3.9.1 according to the method previously described [65]. The ASVs for the intestinal samples were mapped to metabolic reactions using available Genome-Scale Metabolic Models (GSMMs) of human gut microbiota [66] as recently described [67].

Statistical analyses were conducted in R v4.2.2 [68]. Alpha diversity comparisons between M and V fish and between phases were statistically analysed using the Mann-Whitney-Wilcoxon test (MWW [69]). Composition relative abundance analyses of top taxa were carried out for M vs. V fish at different phase comparisons using MWW. Type I error rates were controlled using the Benjamini–Hochberg method (BH [70]), at *p* < 0.05. For comparing beta diversity estimates, permutational multivariate analysis of variance (PERMANOVA) and permutation test for homogeneity of multivariate dispersions (PERMDISP) were performed using vegan (v2.6-4 [71]) and pairwise PERMANOVA was analysed by EcolUtils (v0.1 [72]), where *p* < 0.05 was considered significant. Statistical analysis of metabolic reaction analysis was carried out using a two-sample t-test in Python 3.9.1 using the ttest_ind function (SciPy module v1.9.3 [73]), to compare the mean abundances of each metabolic reaction for M vs. V fish and adjusted using the BH method to correct for multiple testing. The metabolic pathway classification of reactions was obtained from the GSMMs, and Fisher’s exact test was used to identify enriched pathways among the significantly different reactions. The pathways with BH-adjusted *p* ≤ 0.05 were regarded to be enriched. Further, principal component analysis (PCA) was performed separately on standardised ASVs and reaction abundances (z-scores) to see whether any of the abundances could explain the variability in the data.

## Results

### Fish performance

At the end of stimulus stage, V fish showed lower specific growth rate (5.9%/day) than M fish (6.3%/day), whereas at intermediate, M and V fish showed comparable specific growth rates (4.0%/day), feed efficiencies (M fish 1.4, V fish 1.3) and survival rate (M fish 97.9%, V fish 97.3%). Similarly, after the V challenge phase, comparable specific growth rates (1.8%/day), feed efficiencies (1.0) and survival rate (M fish 100%, V fish 99.6%) was revealed in M and V fish (for full details regarding feeding trial and fish see McMillan et al. [51]).

### Reads and ASVs of sequenced data

After sequence denoising and ASV filtering and clustering, a total of 7.8 million reads were retained for the downstream data analysis for intestinal (5.5 million reads), feed (0.7 million reads) and water (1.5 million reads) samples. The median number of reads across samples used for downstream analysis was 24,732, with the lower quartile of 7,633 and upper quartile of 76,326 reads, respectively. The reads for the downstream analysis generated a total of 4,988 unique ASVs (all assigned down to family level), of which 78.5% were assigned at the genus level and 9.7% at the species level.

### Alpha diversity

**Fig. 2:**
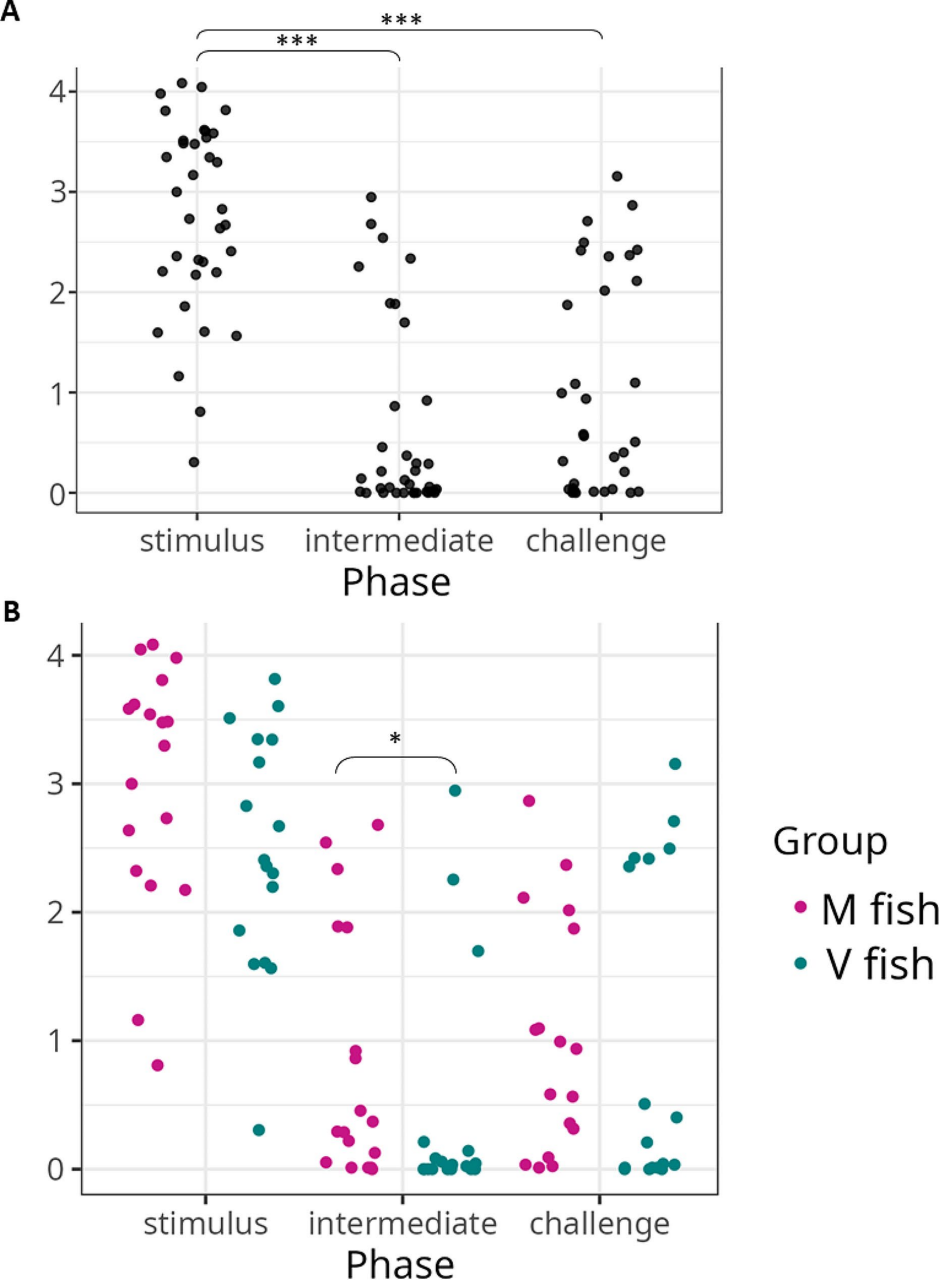
Shannon-Weiner estimate of alpha diversity of the intestinal microbiota of fish fed the experimental diets at different phases (**A** and **B**) and across all phases (**C**). Statistical significance (MWW-tested and BH-corrected) is shown represented by * (*p* < 0.05), *** (*p* < 0.001) between different groups and treatments (*n* = 18/treatment)

The intestinal microbiota in the stimulus phase regardless of the fish group was significantly higher in the Shannon-Weiner estimate of alpha diversity than in the intermediate and challenge phases (Fig. 2A). Also, M fish showed significantly higher alpha diversity than V fish (M = 0.83, V = 0.45) at the intermediate phase sampling with no statistical difference shown at stimulus (M = 3, V = 2.5) or challenge (M = 1.01, V = 0.98) phases (Fig. 2B and Table S2).

M and V feed samples showed no significant difference in alpha diversity (Table S2). No differences were detected for water samples collected from M and V tanks at each phase or between phases (Table S2). When comparing different sample types, the intestine demonstrated a significantly lower Shannon-Weiner than water and feeds (Table S2).

### Beta diversity

**Fig. 3:**
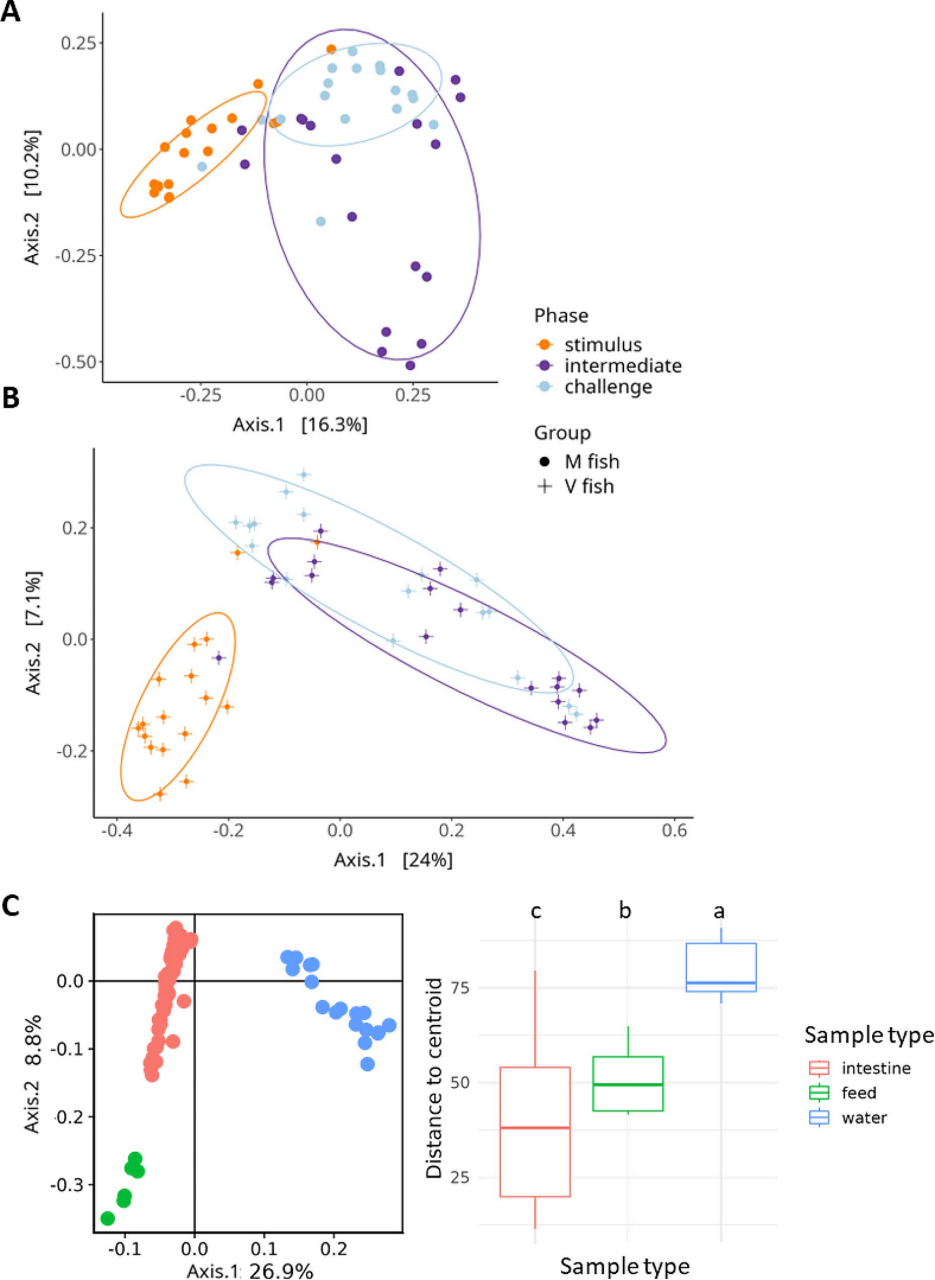
PCoA of the beta diversity of microbiota in the intestine (Bray-Curtis distances) of M fish (**A**), V fish (**B**), and intestine, water, and feed (robust Aitchison distances) (left panel of **C**). The statistical significance of the permutation test for homogeneity of multivariate dispersions (PERMDISP) shows water is the highest and intestine is the lowest (right panel of **C**)

Beta diversity was assessed using PCoA based on BC (Fig. 3) and PERMANOVA analyses to investigate microbiota dissimilarity. PERMANOVA general effects revealed microbiota dissimilarity between gut by fish group (V vs. M fish) and phases (stimulus vs. intermediate vs. challenge). However, no interaction effect of both groups and phases was demonstrated (Table 1). At stimulus and challenge stages, the Bray–Curtis distance indicated no significant differences in microbiota between M and V fish whereas they were significantly different at the intermediate stage (Table 1, PERMANOVA *p* < 0.01, PERMDISP *p* > 0.05).

**Table 1:** PERMANOVA (global and pairwise) analysis (999 permutations) on Bray-Curtis distances for beta-diversity of microbiota comparisons in Atlantic salmon grouped by fish groups or phase for intestinal samples (*n* = 18/treatment). For sample type comparisons (intestine *n* = 105, water or feed *n* = 3), PERMANOVA analysis was carried out on robust Aitchison distances. SumsOfSqs: sum of squares

Comparisons		SumsOfSqs	F.Model	*R* ^2^	Corrected *p*-values
General effects	group	0.541	1.998	0.016	**0.013**
	phase	4.621	8.525	0.141	**0.001**
Interaction effects	group: phase	0.655	1.208	0.020	0.142
M vs. V fish	at stimulus	0.2	0.82	0.024	0.832
	at intermediate	0.697	2.46	0.068	**0.007**
	at challenge	0.283	0.99	0.03	0.451
Stimulus vs. intermediate	M fish	1.682	6.11	0.152	**0.002**
	V fish	2.118	8.37	0.202	**0.002**
Stimulus vs. challenge	M fish	1.416	5.33	0.139	**0.002**
	V fish	1.568	5.95	0.157	**0.002**
Intermediate vs. challenge	M fish	0.682	2.34	0.066	**0.003**
	V fish	0.443	1.60	0.046	0.076
M vs. V water	at stimulus	0.074	0.86	0.176	0.7
	at intermediate	0.047	0.83	0.172	1
	at challenge	0.065	0.90	0.183	0.467
feed vs. intestine		33,020	0.14	0.001	**0.003**
feed vs. water		69,592	0.34	0.001	**0.003**
intestine vs. water		114,325	0.28	0.001	**0.003**

PCoA (Fig. 3A, B) and PERMANOVA analyses showed significant differences in microbiota between stimulus and both intermediate and challenge (*p* < 0.01) in any of the fish groups. Unlike V fish (Fig. 3B), M fish microbiota at intermediate was dissimilar from challenge by PCoA and PERMANOVA (*p* < 0.01) analyses, which is not attributed to the variability in samples (PERMDISP *p* > 0.05). The microbiota of water samples showed no PERMANOVA dissimilarity between M and V water (Table 1). Robust Aitchison distance-based phylogenetic beta diversity showed a clear separation of each of the intestine, feed and water microbiota (Fig. 3C, PERMANOVA *p* < 0.01), while PERMDISP showed the highest dispersion in water samples, and the least was in intestinal samples (Fig. 3C).

### Intestine, feed, and water-associated microbiota

**Fig. 4:**
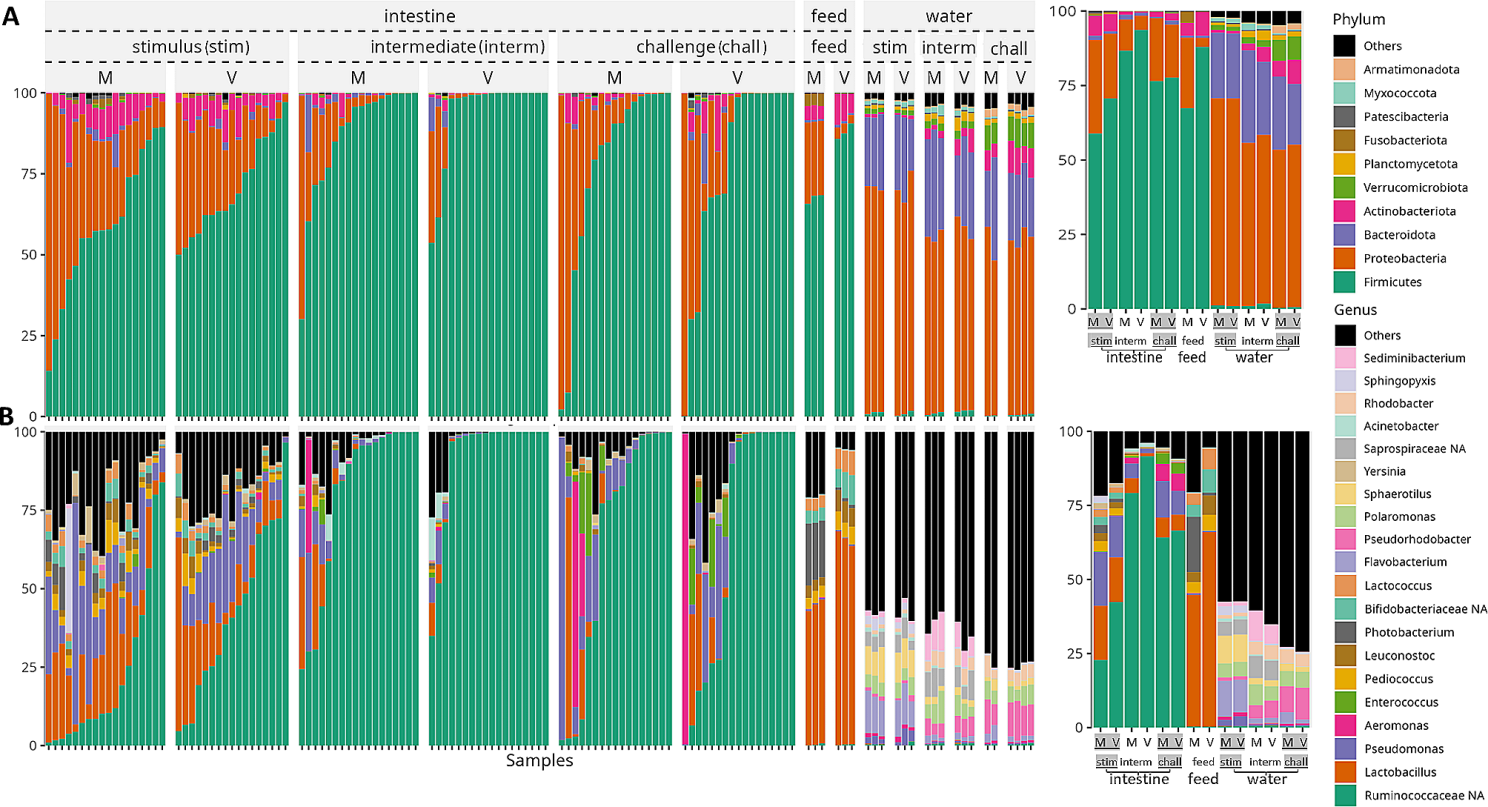
Relative abundance (as % from 100% total on the y-axis) of the microbiota of the intestine, water, and feed associated with different dietary groups in response to M vs. V diets at phylum (**A**) and genus (**B**) levels. Individual and grouped (averaged) samples are presented on the left and right panels, respectively

**Fig. 5:**
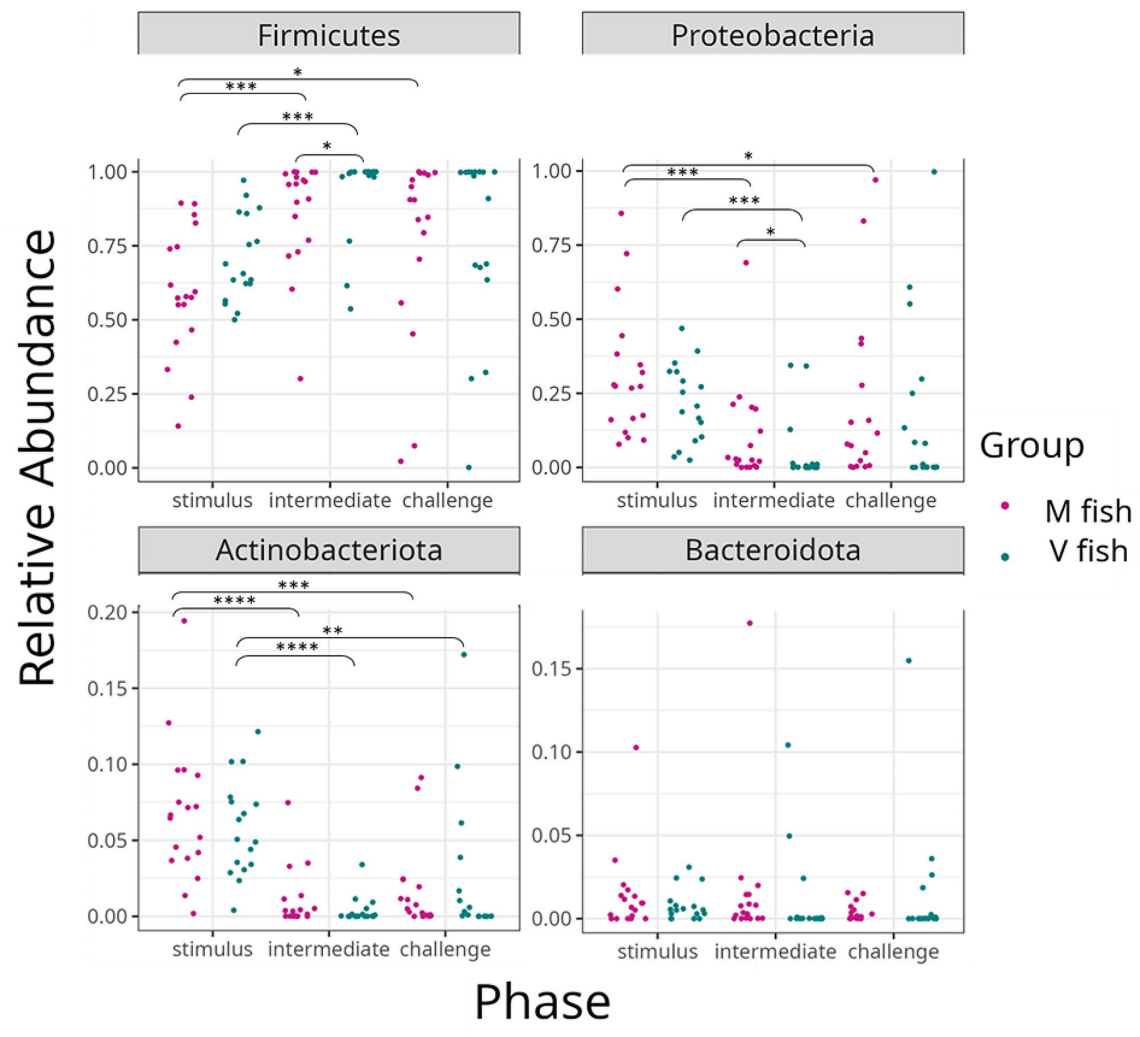
Relative abundance of the top four most abundant phyla in the intestine of salmon fed the experimental diets. The samples are grouped by phases, and the fish dietary group and significant difference (MWW-tested and BH-corrected) is represented by * (*p* < 0.05), ** (*p* < 0.01), *** (*p* < 0.001), **** (*p* < 0.001)

Regardless of the fish groups and phases, the taxonomic compositions of the intestinal samples at the phylum level were dominated by the phyla Firmicutes, Proteobacteria, Actinobacteriota and Bacteroidota (Figs. 4A and 5, Table S3). Firmicutes relative abundance (RA) averaged 67.5% at stimulus, which increased at intermediate (averaged 90%) and then declined at the challenge (averaged 76.5%). Proteobacteria RA showed an opposite pattern where RA averaged 24% at the stimulus that declined at the intermediate (averaged 7.5%) and then increased at the challenge (19.5%). The top two phyla RA significantly differentiated in M vs. V fish [Firmicutes (V > M), Proteobacteria (M > V)] at intermediate phase (Fig. 5). Proteobacteria at stimulus was higher than intermediate and challenge (Fig. 4A). The top three taxa at genus level or lowest identified taxonomic rank are undefined genera of family Ruminococcaceae, genera *Lactobacillus* and *Pseudomonas* respectively (Fig. 4B, Table S4). The top 15 taxa and ‘OTHERS’ (all other taxa after the top 15, Table S4) showed higher RA in stimulus than intermediate and challenge for either M or V fish with some exceptions (Table S4). M and V fish shared the highest ASVs share at stimulus (20% of the total ASVs) than later stages (intermediate 15%, challenge 17%) (Fig. S3).

**Fig. 6:**
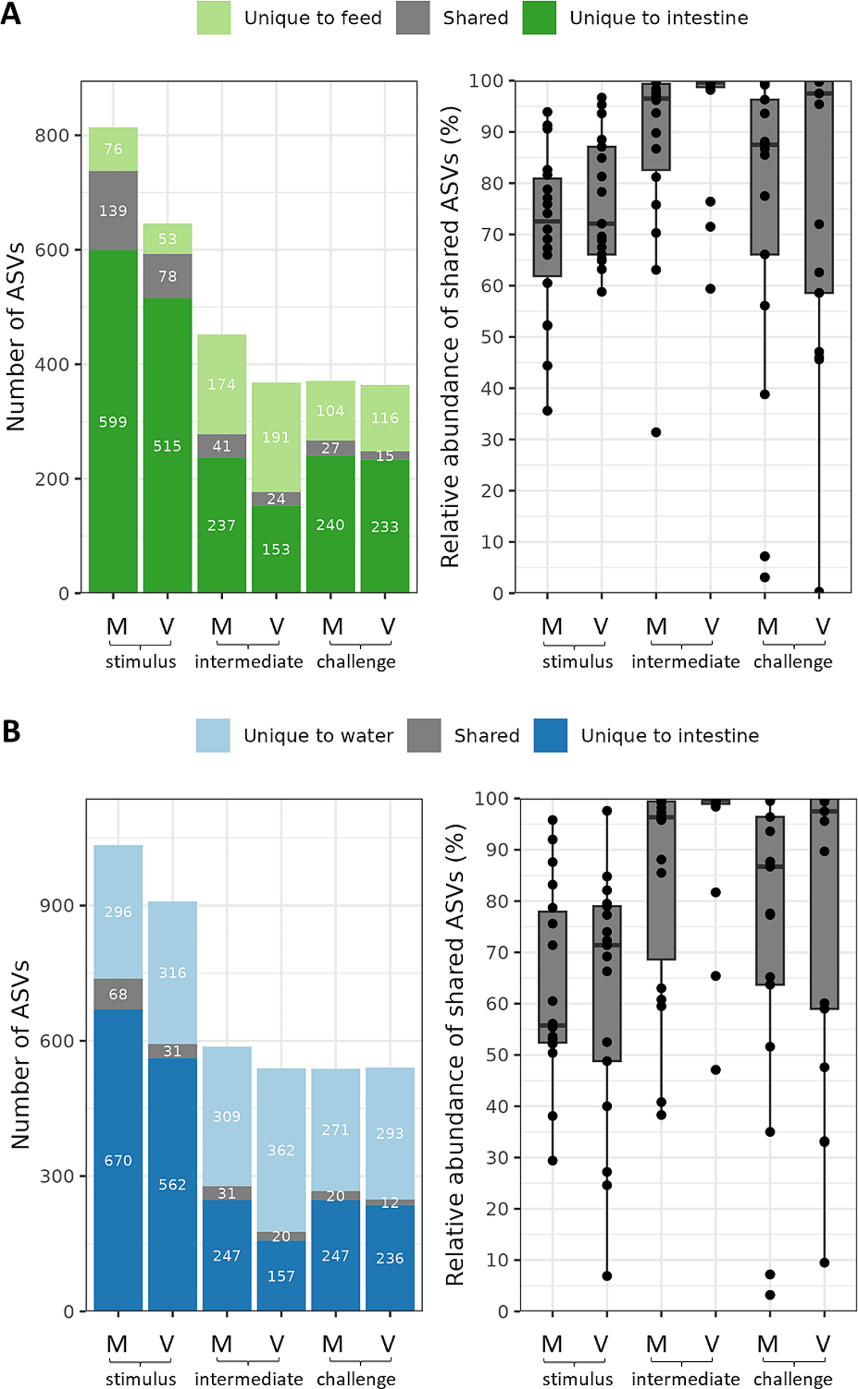
The shared ASVs numbers and relative abundance between the intestine and feed (**A**) and between intestine and the water (**B**). ASVs with minimum relative abundance in a sample of 0.05% were included. The M and V fish are labelled across the phases (stimulus, intermediate and challenge)

At the phylum level (Fig. 4A), the feed-associated microbiota was dominated by the same top phyla: Firmicutes [where V diet (88%) > M diet (67%)] and Proteobacteria [M (24%) > V diet (3%)]. At genus (Fig. 4B), *Lactobacillus* (62–65%) dominated both diets while *Photobacterium* was of higher abundance in M (19%) than V diet (0.97%). The microbiota in the tank water was dominated by phyla Proteobacteria and Bacteroidota (Fig. 4A). For water accompanying different fish groups, top taxa at genus level were different from those of intestine and feed (Fig. 4B). The number of intestinal ASVs shared with either feed or water at stimulus was higher than during intermediate or challenge phases (Fig. 6). Intestinal ASVs share with feed was higher than with water across phases (Fig. 6).

### Metabolic reaction analysis of microbiota

Of the 2,150 ASVs, 1,368 could be mapped to at least one GSMM. Among these, 617 were matched to family with an average of 10 models per ASV, 665 were matched to genus with an average of 17 models per ASV, and 86 were matched to species with an average of 1 model per ASV (Fig. S4). In total, the models mapped to ASVs contained 4,817 different reactions; most of these reactions (78%) were present in all samples, and all samples contained at least 79% of the reactions. Most samples (95%) contained more than 99% of the reactions, but the abundances of many reactions differed significantly between samples and fish groups. Furthermore, PCA of reaction abundances allowed much more variability in the data to be explained than PCA of ASV abundances (Fig. S5, Fig. S6).

**Fig. 7:**
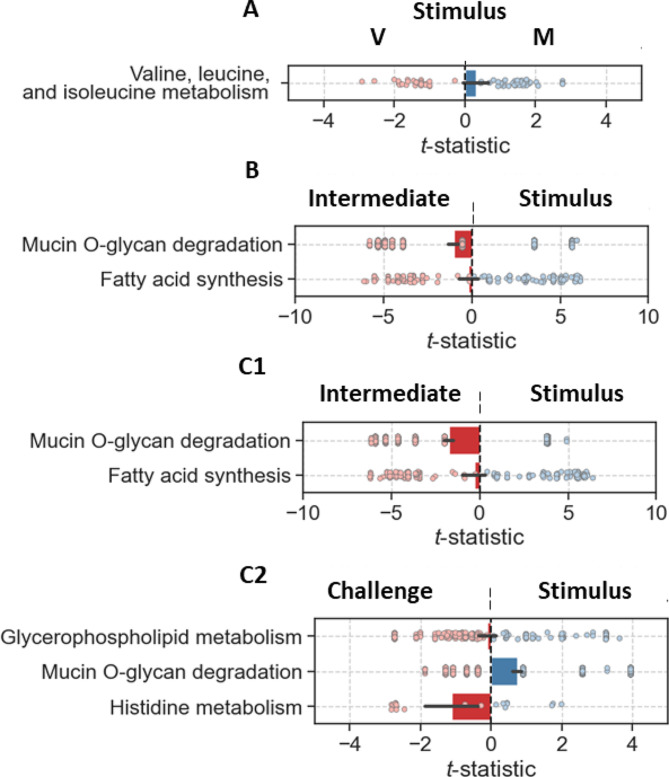
Metabolic reactions analysis using t-tests comparing reaction abundances between (A) M vs. V fish at each phase, (B) M fish group and (C) V fish group. The t-statistic for each reaction and the mean across all reactions with a 95% confidence interval for all significantly enriched subsystems

Grouping reactions by metabolic pathways, we found that five pathways were enriched in reactions with significantly different mean abundances between fish groups (Fig. 7). For developmental stages comparisons, the intestinal microbiota of M fish at stimulus showed predicted enrichment of metabolic pathways related to valine, leucine, and isoleucine metabolism compared with V fish (Fig. 7A). There was no significantly different predicted enrichment of metabolic pathways between fish groups at either intermediate or challenge phase.

The intestinal microbiota of both fish groups showed enriched pathways at stimulus compared to intermediate (mucin O-glycan degradation and fatty acid metabolism) (Fig. 7B, C1). V fish at challenge showed lower mucin O-glycan degradation and higher glycerophospholipid and histidine metabolism enrichment than stimulus (Fig. 7C2).

## Discussion

Nutritional programming has been employed to reduce the adverse effects of plant-derived dietary ingredients in commercial Atlantic salmon feed formulations, but the mechanisms behind NP effects, required to determine appropriate intervention timing and delivery methods, are as yet unclear [25, 46]. Previous studies investigated whether NP modulated the gut microbiota and if such modulation could be coupled with fish performance [25, 46–49]. In this study, we compared the effects of first feeding with a plant-based diet vs. a traditional marine-based diet on the developmental microbial composition and diversity of the intestine in Atlantic salmon. Although there were limited effects on growth [51], we found that the V diet induced significant changes in the gut microbiota. Interestingly, persistent microbial changes were identified in the gut microbiota throughout the experiment in the V nutritionally programmed group but not the M fish group. This highlights potentially important interactions between gut microbiota and the V diet and suggests possible microbial mechanisms underlying NP. Amino acid metabolism pathways were enriched in the M fish as an example of microbiota involvement in aiding digestion while the excessive mucin O-glycan degradation was mitigated after NP.

### Environmental and host factors contribute differently to microbiota composition across development

Water and feed are the primary exogenous gut microbiota sources [74]. Feed is well-known to impact the gut microbial composition of fish (wild and laboratory), mice and humans [75]. Microbial inoculation from water starts even before the onset of feeding, as a NP study on zebrafish gut revealed similar microbiota between individuals after hatching [49]. Our study showed a decrease in the richness and diversity of gut microbiota over time (from stimulus to challenge), which might reflect a higher diversity at the time of colonisation (dispersal phenomena; [76]). A reduction in richness and diversity of the gut microbiota throughout Atlantic salmon ontogeny from embryo through first feeding [7–8 weeks post-hatching (wph)] and up to seawater adult developmental stages has been identified [77], which is in line with our pattern of decreasing richness and diversity. Similarly, greater richness was found in the gut microbiota of zebrafish juveniles than in adults [76, 78]. The number of intestinal ASVs shared with either feed or water at stimulus was higher than during intermediate or challenge phases which might suggest that fish select their microbiota from those in the environment at early developmental stages. In line with this theory of selection and dispersal [76], stimulus, among all phases, had the highest ASV share (20%) between the fish groups (M and V fish), despite being fed different diets. Selection and dispersal are the main examples of deterministic processes suggested to happen at larval/early developmental stages in different fish trophic levels (including carnivores) [76]. On the other hand, drift is the primary suggested process (a stochastic mechanism) that happens in later development and adult stages and enhances the filtering of particular community species regardless of the common microbiota in the surrounding environment [76]. No microbiota dissimilarity was found between M and V fish at stimulus, suggesting that at the early life stage (two wpff), the environmental factor is the primary determinant factor for the gut microbiota assemblage. As fish develop, in this case at intermediate and challenge phases, their gut microbiota differentiate from the stimulus intestinal microbiota. Similarly, larval fish gut-associated communities were more similar to the surrounding environmental communities than were in adults, as previously reported in zebrafish [79]. The developmental stage is one of the most critical (host) factors [78] along with environmental factors to shape the gut microbiota at early development that is likely to reflect the gut development. Similarly, genotype/genetics (genetically improved vs. reference fish) as a host factor in European sea bass (*Dicentrarchus labrax*) had a higher influence in modulating gut microbiota than diet (FM-based control diet vs. diet based on poultry meal and oil with microalgae oil) [80]. In a similar study on gilthead sea bream (*Sparus aurata*) using similar diet, genetics had a long-term effect on gut microbiota with decreased impact in later developmental stages during 12-month production cycle from juvenile to adult stages regardless of the diet [81]. It is worth noting that the sampled intestine region (whole at stimulus compared to distal at intermediate and challenge) may be a contributing factor to the microbiota dissimilarity across developmental stages that requires further research. Nevertheless, microbial composition showed no overlap between stimulus whole intestine and later stages distal intestine suggesting that the microbiota dissimilarity might highly not involve gut sections difference although the stimulus whole gut contains distal intestine. Moreover, the intestinal mucosa or digesta showed differential microbial composition in Atlantic salmon on post-smolts kept in RAS for four weeks in response to commercial diet (43% plant and 57% marine ingredients) [82] and on 16-week seawater feeding trial regardless of diet (commercial vs. insect-based) [83]. Thus, how mucosa and digesta responded to M or V diet particularly during first feeding, requires further study since herein the collected samples are both mucosa and digesta.

### Nutritional history is a key factor in microbial diversity at later developmental stages

Studies of NP with a similar experimental design to what we have described here have shown no microbiota difference throughout the feeding trial and thus concluded that NP had not impacted the gut microbiota [45, 46, 49]. These studies proposed that the environmental factors represented by the RAS water tank abiotic conditions, including pH, temperature, and salinity, may outweigh the effects of NP and dietary protein sources. However, in phases of the current study, M and V fish showed significant dissimilarity. Other studies also suggested that host-related factors might be the reason for the similarity across sampling points and dietary fish groups rather than environmental factors [45, 46, 49]. In the current study, the intestinal microbiota showed higher richness and diversity for M than V fish and dissimilarity in the intermediate phase, although they were fed the same M diet at this phase, suggesting that the feed history at stimulus may have acted as the primer. However, during the stimulus phase, M fish showed a similar richness and diversity compared to V fish, with no microbiota dissimilarity, although fish were fed different diets. This similarity might be due to the high microbial acquisition from the surrounding environment at this developmental stage. Thus, it could be implied that the first feeding history could be considered a determining factor in the gut microbiota composition in later life. Furthermore, from a NP perspective, PERMANOVA analysis showed microbiota dissimilarity in the M fish between intermediate and challenge but not in the V fish. These results suggest that although the fish in both groups were fed the M diet followed by the V diet, V fish were programmed and adapted their gut to selectively choose a particular microbial assemblage probably due to nutritional history even after interruption of V feed continuity by M diet (i.e., programming effect). Similarly, rainbow trout showed that diet (M vs. V) type fed at first feeding significantly determined the gut microbiota at the later developmental stage (7 wpff) [84]. Stimulus microbiota dissimilarity from intermediate and challenge regardless of fish dietary group/history is expected, as discussed before (i.e., stage-specific community assemblage). While for M fish, the microbiota was found to be different at each phase, suggesting the intestinal microbiota was impacted in a flexible manner with fish development (stimulus vs. intermediate) and with dietary change (intermediate vs. challenge). Similarly, Atlantic salmon gut microbiota is reported to be highly affected by diet (i.e., non-programmed fish) [85]. These indications likely reflect that the early NP regime of dietary plant exposure at stimulus impacted gut microbiota.

### NP impact on functional capacity of microbiota

We hypothetically propose that the complex nature of how plant-derived feeds interact with the intestine in fish is likely to be influenced by the intestinal microbiota, which can aid in both digestion of plant-derived molecules and production of metabolites beneficial to the intestinal cells. Several different mechanisms could explain the microbiota’s involvement. Firstly, microbes that aid digestion are retained at a low level and then re-establish quicker during re-exposure to the feed. Secondly, microbes present at first feeding reappear later in life, and the intestine can tolerate these bacterial species and their metabolites, allowing the programmed fish to perform better. Thirdly, metabolites generated by bacterial species can induce intestinal gene expression of the fish to utilise better the plant materials, which requires further metabolic and transcriptomic studies. Degradation and fermentation of complex carbohydrates as one of the functions of microbiota have been studied in terms of how it induces host immune responses (as reviewed by [86–90]).

We investigated the predicted microbial metabolic reactions and found that the microbiota showed enriched valine, leucine, and isoleucine (essential amino acids) metabolism in M fish compared to V fish at stimulus, suggesting that the microbiota can contribute to the metabolism of essential amino acids found in the M diet. Amino acid metabolism was improved in the gut microbiota of common carp (*Cyprinus carpio*) fed ryegrass vs. the commercial feed [91]. A study on mammalian genotype impact on the faecal microbiota showed enrichment of enzymes that map to nine amino acid degradation in carnivores vs. 12 amino acid biosynthesis pathways in herbivores [92]. From a NP perspective, mucin O-glycan (complex carbohydrate) degradation was enriched at stimulus when compared to challenge in V fish. Mucin O-glycan is a polysaccharide that mainly composes the mucins that are secreted to protectively line the intestinal epithelium against pathogens or help attenuate their virulence [93] in addition to acting as receptors for microbiota, suggesting that the V diet, especially after first exposure, may increase the degradation of mucin O-glycan which in turn could lead to inflammation. A study on a starch-based diet in piglets showed higher mucin O-glycan degradation compared to an animal-based diet [94]. Mucin O-glycan is the main nutrient for microbiota to generate short-chain fatty acids (SCFAs) essential later on for mucin secretion [95, 96]. As the intestine develops, wear and tear/repair generate mucin O-glycan degradation within acceptable range levels as shown at intermediate when compared to stimulus regardless of the fish dietary group. Furthermore, mucin O-glycan degradation in V fish was enriched at stimulus compared to the challenge phase, which might reflect the programming effect, which supports the third proposal that microbial metabolites have induced intestinal adaptation at the level of gene expression supporting the intricate microbiota-gut interactions. Moreover, although microbiota dissimilarity was noticed at the intermediate stage (as represented by the top phyla and genera), metabolic functional differences were noticed earlier at the stimulus stage, suggesting that some metabolites most probably are carrying out the functionality. On a developmental level, fatty acid synthesis and mucin O-glycan degradation were enriched at the intermediate when compared to stimulus phase, which could be potentially because the M diet at the intermediate has higher lipid and carbohydrate levels than stimulus diets required at this developmental stage. The inferred functionality is difficult to attribute to a specific bacterium as the dissimilarity is represented by microbial assemblage rather than a bacterium.

While 16 S amplicon sequencing provided valuable insights into the microbial diversity in this study, it is essential to acknowledge its limitations in assessing functional capabilities directly as it is based on conserved 16 S rRNA gene (i.e., V3-V4 regions) [97]. The inferred functional potential using the prediction tools thus should be interpreted with caution. Additionally, the current study relies on known GSMM collection of human gut microbiota [66] and is not a full representation of the fish gut microbiota. To comprehensively understand the functionality of the complex microbial assemblages of fish gut and their interactions across the gut-microbiota axis, future studies could incorporate shotgun metagenomics sequencing with the help of metatranscriptomics and metaproteomics, which directly assesses the genetic content (up to species/strain level resolution dependent on sequencing depth) and functional potential of the microbiome [98, 99].

### Concluding remarks

We conclude that the microbiota is likely to play a role in NP mechanisms. Additionally, dietary regime/history influenced the gut microbial structure of the plant-based dietary group with sustained changes after six weeks of challenge with a similar plant-rich diet, whereas environmental and host factors were the prominent influencing factors in the fish initially fed marine diet. Moreover, amino acid metabolism pathways were enriched in the M fish as an example of microbiota involvement in aiding digestion. Mucin O-glycan-microbiota interactions helped understand to some extent how NP could impact the microbiota. Still, future studies are required to explore other possible associations between NP and the gut microbiota. Studies investigating how different NP windows with respect to timing and duration are required in addition to the duration of NP effects can be sustained beyond 22 weeks after first feeding. Also, there is a need to investigate the plant-based diet composition to which Atlantic salmon and other species have higher sensitivity during their plastic developmental stages to elicit NP effects. Analysis of the gut microbiota on a species level is also recommended to closely identify species functions and detect more of the possible diet-microbiota-gut axis interactions.

### Electronic supplementary material

Below is the link to the electronic supplementary material.

Supplementary Material 1

Supplementary Material 2

Supplementary Material 3

Supplementary Material 4

Supplementary Material 5

Supplementary Material 6

Supplementary Material 7

## Data Availability

The raw 16S rRNA gene sequence data and metadata files are deposited at the NCBI SRA database under the BioProject PRJNA1054346 at link https://www.ncbi.nlm.nih.gov/bioproject/PRJNA1054346 (BioSample DNA accession numbers: SAMN39273568-704). MMT’s created Rcode that was used in the data analysis and made available in the Github repository at link https://github.com/marwa38/NP_devStages_ampliseq.git for reproducing the results.

## Abbreviations

ASVs: Amplicon Sequence Variants
F: Forward reads
FM: Fishmeal
FO: Fish oil
GSMMs: Genome-Scale Metabolic Models
LC-PUFA: Long-chain polyunsaturated fatty acids
M: Marine-based
NP: Nutritional programming
PCA: Principal Component Analysis
PCoA: Principal Coordinates Analysis
PERMANOVA: Permutation Multivariate Analysis of Variance
PERMDISP: Permutation test for homogeneity of multivariate dispersions
R: Reverse reads
RA: Relative abundance
RAS: Recirculating aquaculture system
SEM: Standard error of the mean
SPC: Soy protein concentrates
V: Plant/vegetable-based
wpff: Weeks post-first feeding
wph: Weeks post-hatching
