# Supplementary figures and images for: Modulation of gut microbiota composition and predicted metabolic capacity after nutritional programming with a plant-rich diet in Atlantic salmon (Salmo salar): insights across developmental stages
Source: Anim Microbiome. 2024 Jul 1;6:38. doi: 10.1186/s42523-024-00321-8 (PMC11218362; doi:10.1186/s42523-024-00321-8)

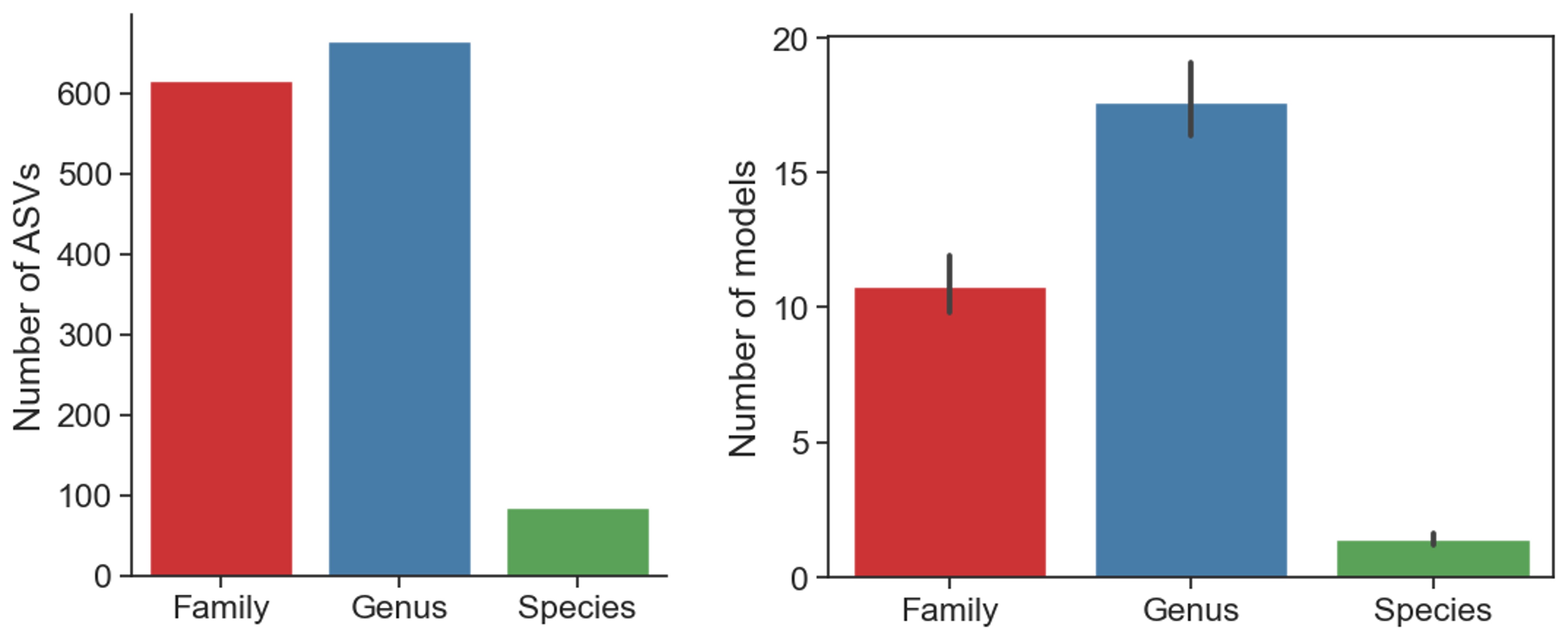

Supplement: Supplementary file 1 — Supplementary Material 1 [file 42523_2024_321_MOESM1_ESM.jpg]

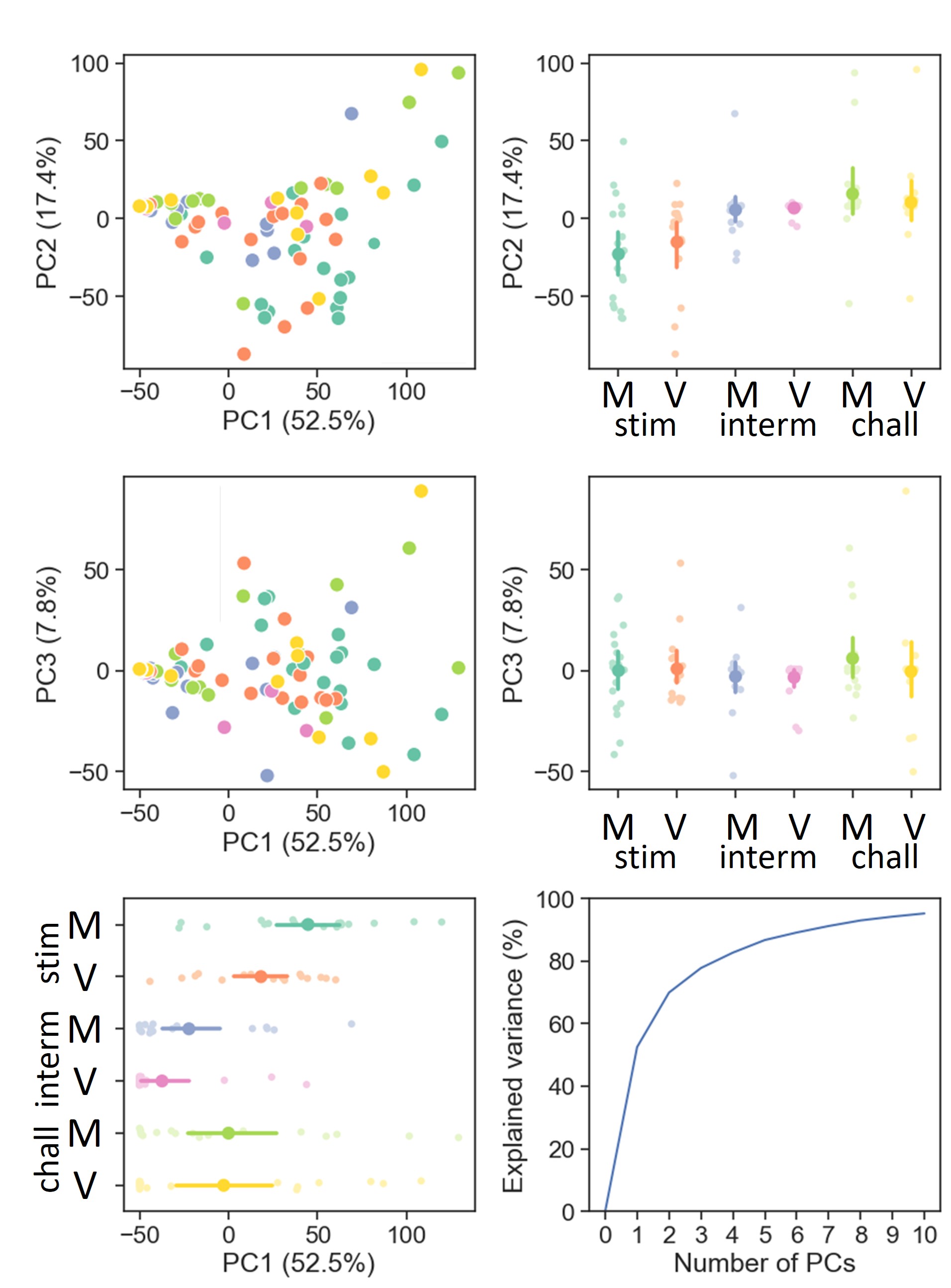

Supplement: Supplementary file 2 — Supplementary Material 2 [file 42523_2024_321_MOESM2_ESM.jpg]

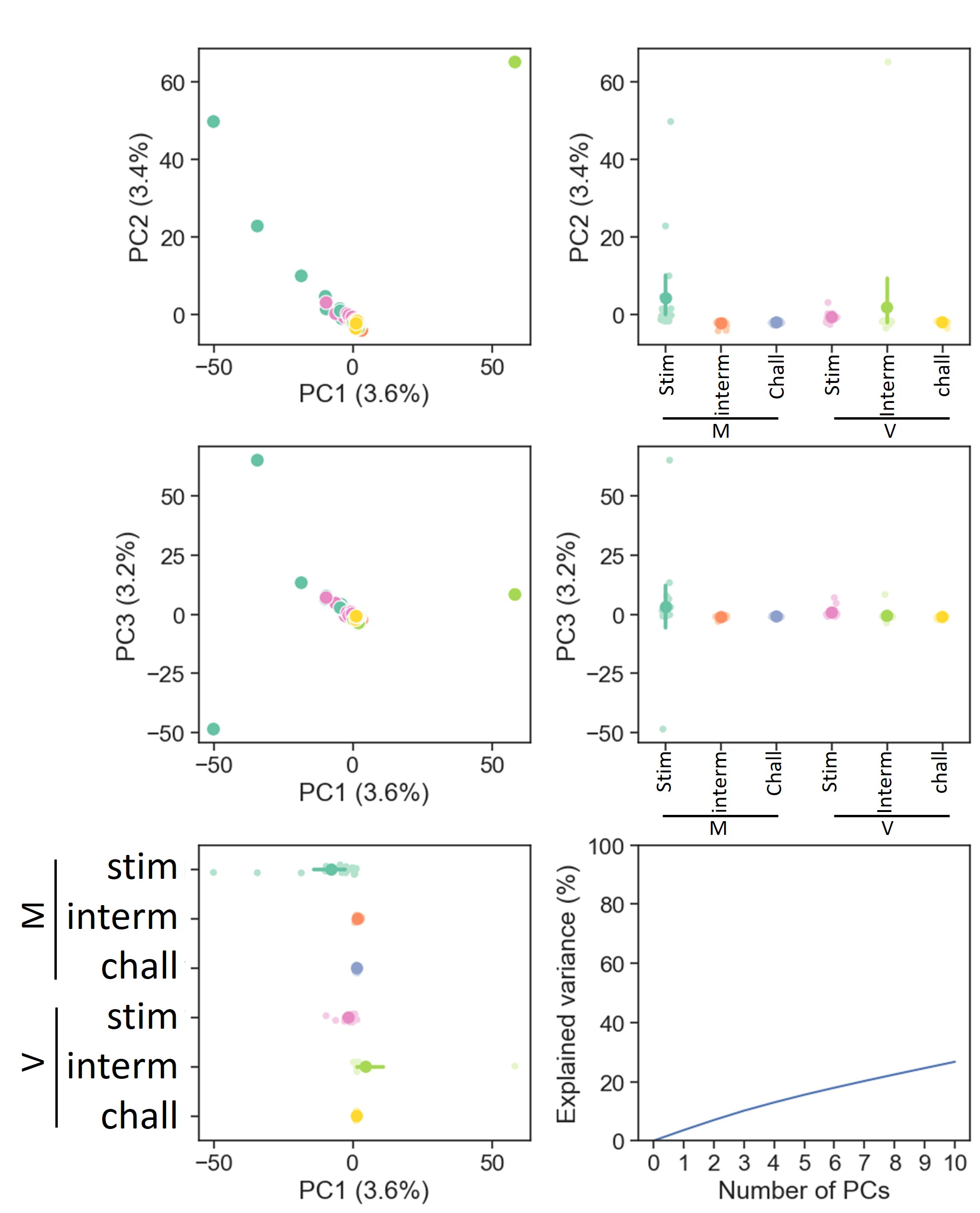

Supplement: Supplementary file 3 — Supplementary Material 3 [file 42523_2024_321_MOESM3_ESM.jpg]

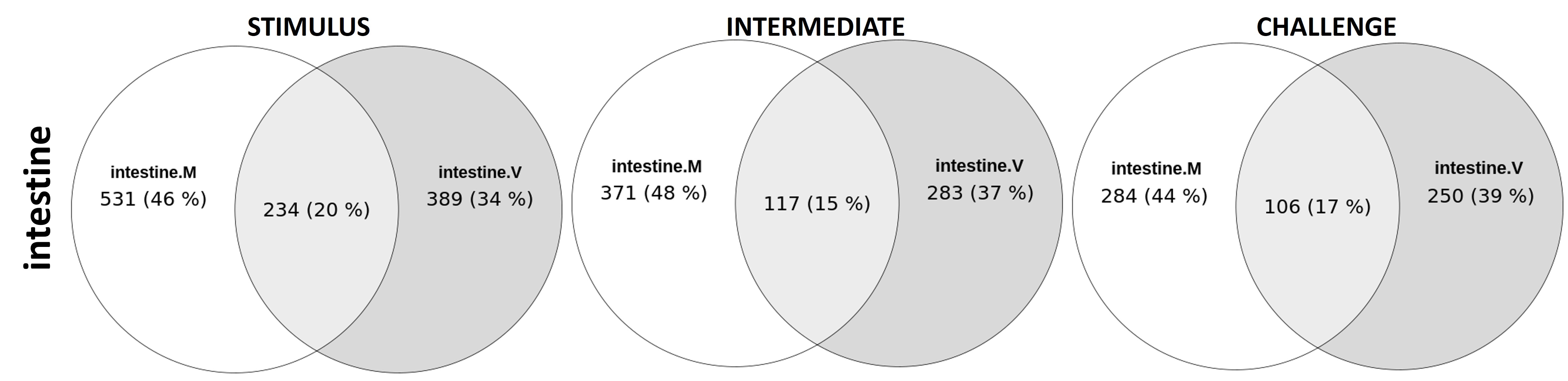

Supplement: Supplementary file 4 — Supplementary Material 4 [file 42523_2024_321_MOESM4_ESM.jpg]

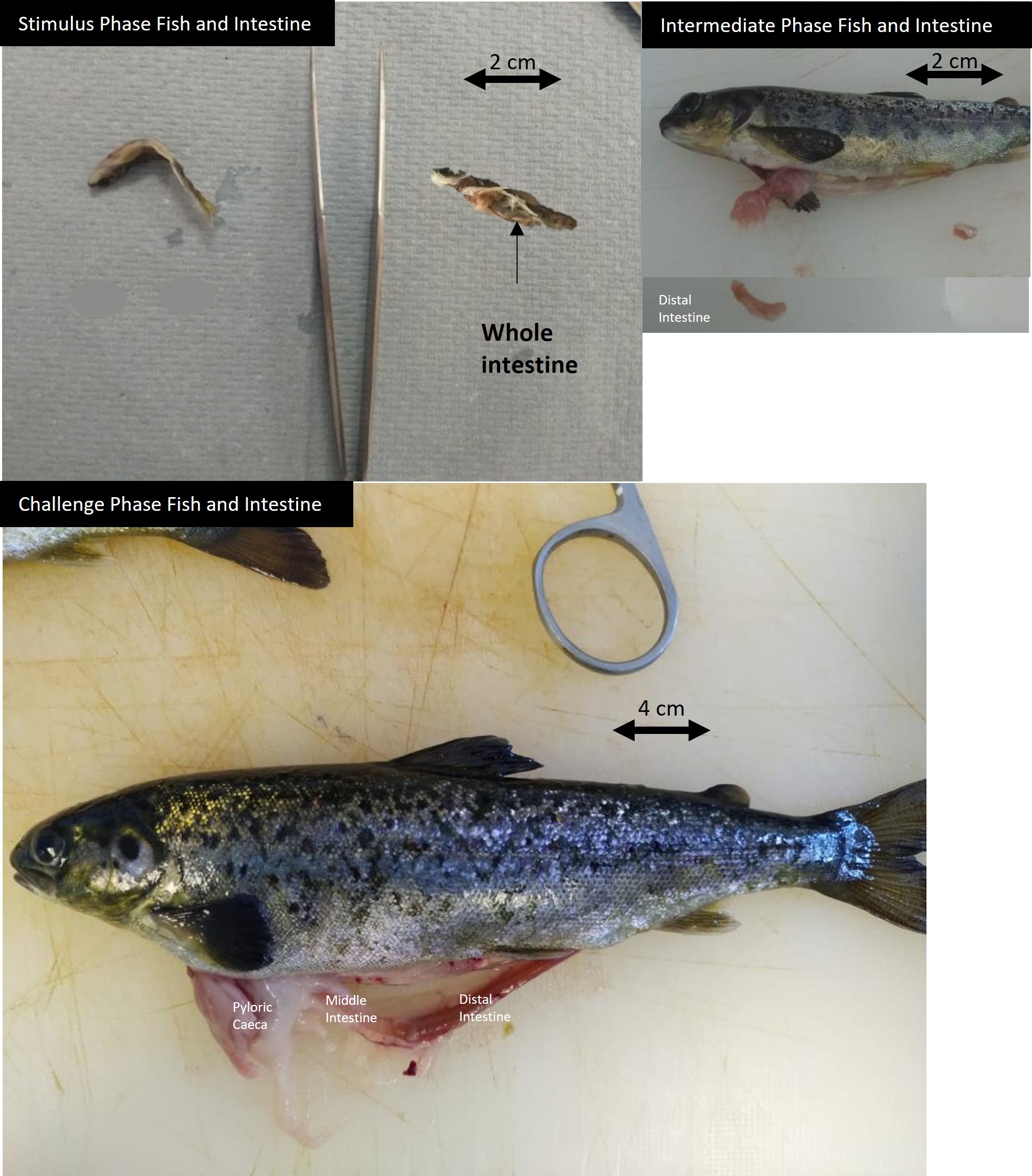

Supplement: Supplementary file 5 — Supplementary Material 5 [file 42523_2024_321_MOESM5_ESM.jpg]

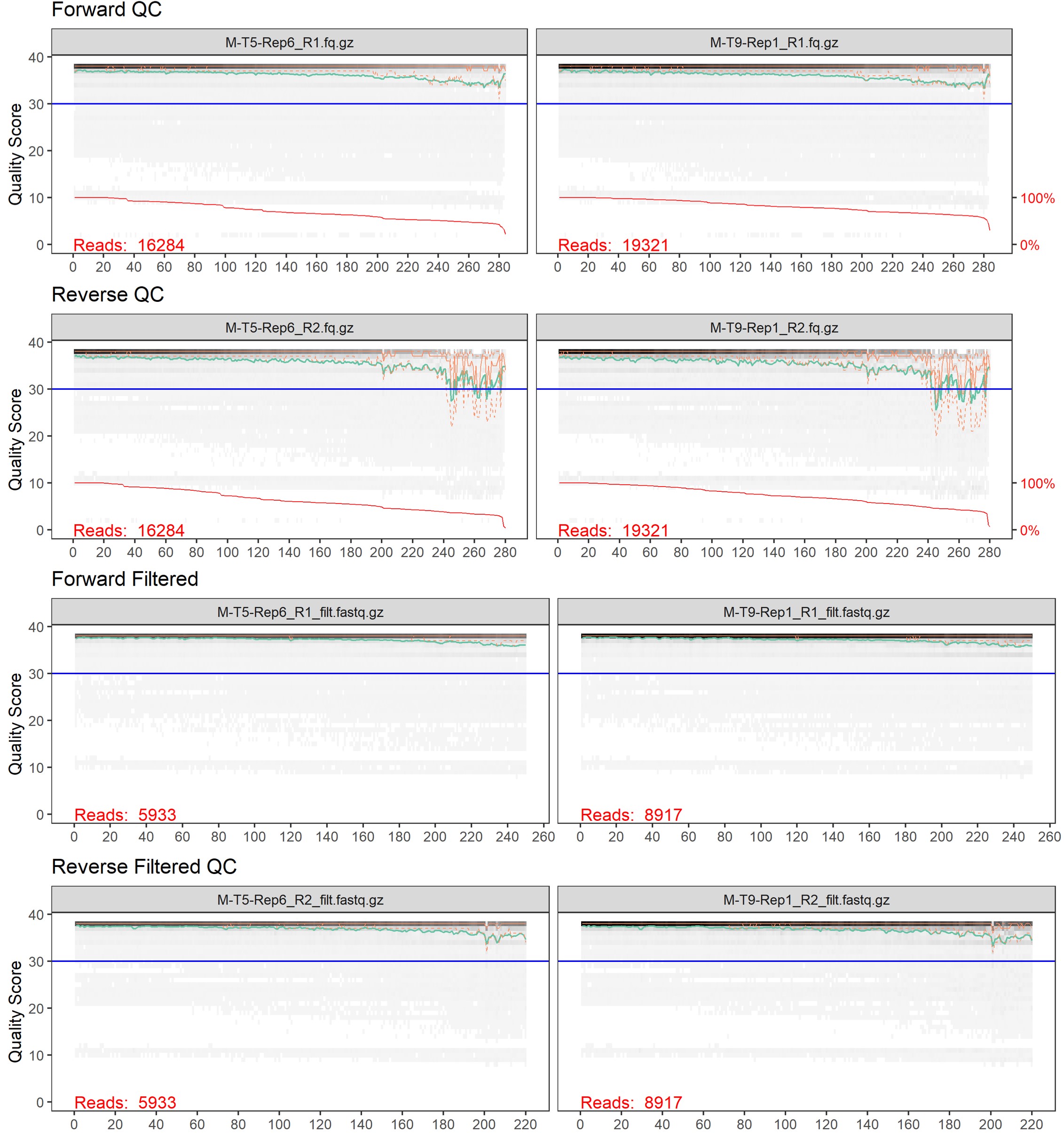

Supplement: Supplementary file 6 — Supplementary Material 6 [file 42523_2024_321_MOESM6_ESM.jpg]
