# Supplementary material for: Modulation of gut microbiota composition and predicted metabolic capacity after nutritional programming with a plant-rich diet in Atlantic salmon (Salmo salar): insights across developmental stages
Source: Anim Microbiome. 2024 Jul 1;6:38. doi: 10.1186/s42523-024-00321-8 (PMC11218362; doi:10.1186/s42523-024-00321-8)
Supplement: Supplementary file 7 — Supplementary Material 7 [file 42523_2024_321_MOESM7_ESM.docx]

1. **Supplementary Information**
   1. **Supplementary Tables**

**Table S1**. Formulation, proximate and fatty acid compositions of standard marine diets (M_S_ and M_I_) and low fishmeal/fish oil vegetable-based diets (V_S_ and V_C_) used in respective feeding phases.

ALA, α-linolenic acid; ARA, arachidonic acid; LA, linoleic acid; LC-PUFA, long-chain polyunsaturated fatty acid; MUFA, monounsaturated fatty acid; OA, Oleic acid; PPC, pea protein concentrate; SFA, saturated fatty acid; SPC, soya protein concentrate.

* Feed Services Bremen, Bremen, Germany.

^†^ Aker BioMarine, Lysaker, Norway.

^‡^ Caramuru, Itumbiara, Brazil.

^§^ Cargill, Minnesota, US.

^||^ Agrident, Amsterdam, Netherlands.

^**^ ED&F Man, London, UK.

^¶^ Nova Nutriway GmbH, Hamburg, Germany

^††^ DSM, Heerlen, Netherlands.

^‡‡^ Evonik, Essen, Germany.

**Table S2.** Statistical analysis of Shannon-Weiner index of intestine, water and feed comparisons, n denotes to the sample number. p.adj: adjusted p-value. Significant difference (MWW-tested and BH-corrected) is represented by * (p < 0.05) ** (p < 0.01) *** (p < 0.001) **** (p < 0.001). We used the same legend color in figures for different phases.

| Comparison | | 1 | 2 | n1 | n2 | statistic | p.adj |
| --- | --- | --- | --- | --- | --- | --- | --- |
| intestine | Within phase | M | V | 18 | 17 | 203 | ns |
|  |  | M | V | 18 | 18 | 240 | * |
|  |  | M | V | 17 | 17 | 170 | ns |
|  | M fish | M | M | 18 | 18 | 302 | *** |
|  |  | M | M | 18 | 17 | 284 | *** |
|  |  | M | M | 18 | 17 | 121.5 | ns |
|  | V fish | V | V | 17 | 18 | 285 | *** |
|  |  | V | V | 17 | 17 | 232 | ** |
|  |  | V | V | 18 | 17 | 103.5 | ns |
| feed | | M | V | 3 | 3 | 9 | ns |
| water | within phase | M | V | 3 | 3 | 4 | ns |
|  |  | M | V | 3 | 3 | 2 | ns |
|  |  | M | V | 2 | 4 | 7 | ns |
|  | M water | M | M | 3 | 3 | 0 | ns |
|  |  | M | M | 3 | 2 | 4 | ns |
|  |  | M | M | 3 | 2 | 6 | ns |
|  | V water | V | V | 3 | 3 | 0 | ns |
|  |  | V | V | 3 | 4 | 12 | ns |
|  |  | V | V | 3 | 4 | 12 | ns |
| Sample type | | intestine | feed | 105 | 6 | 28 | *** |
|  |  | intestine | water | 105 | 18 | 5 | **** |
|  |  | feed | water | 6 | 18 | 6 | *** |

**Table S3.** Taxonomic composition relative abundances in each of the sample type, phase, and fish group. See taxonomic.composition_devStages in https://github.com/marwa38/NP_devStages_ampliseq to download.

**Table S4.** Top 15 genera (or lowest identified taxonomic rank i.e., family) that showed statistically significant difference (MWW-tested and BH-corrected) between fish groups relative abundances (RA, %). We used the same legend color in figures for different phases. Stars represent corrected statistical significance strength (* p < 0.05, ** p < 0.01, *** p < 0.001, **** p < 0.0001).

| **taxa** | **RA.1** | **1** | **2** | **RA.2** | **p.adj** | **taxa** | **RA.1** | **1** | **2** | **RA.2** | **p.adj** |
| --- | --- | --- | --- | --- | --- | --- | --- | --- | --- | --- | --- |
| Ruminococcaceae*** | 91.6 | V | V | 42.4 | **** | *Lactobacillus* | 18.3 | M | M | 5.025 | ** |
|  | 79.2 | M | M | 22.8 | **** |  | 18.3 | M | M | 6.759 | ** |
| OTHERS**** | 22.2 | M | M | 5.55 | ** |  | 15.1 | V | V | 1.036 | *** |
|  | 22.2 | M | M | 4.67 | *** |  | 15.1 | V | V | 5.397 | ** |
|  | 14 | V | V | 3.5 | *** | *Pediococcus* | 3.39 | M | M | 0.302 | *** |
| *Pseudomonas* | 18.2 | M | M | 4.92 | ** |  | 3.39 | M | M | 0.14 | *** |
|  | 13.9 | V | V | 1.47 | **** |  | 2.33 | V | V | 0.114 | **** |
| *Yersinia* | 1.85 | M | M | 0.2 | ** |  | 2.33 | V | V | 0.001 | **** |
|  | 1.08 | V | V | 0.1 | * | *Photobacterium* | 2.77 | M | M | 0.015 | *** |
| *Staphylococcus* | 1.23 | V | V | 0.1 | ** |  | 2.77 | M | M | 0 | *** |
|  | 1.23 | V | V | 0.3 | * |  | 1.22 | V | V | 0.048 | *** |
| *Cutibacterium* | 1.53 | V | V | 0.11 | **** |  | 1.22 | V | V | 1E-04 | *** |
|  | 1.53 | V | V | 0.16 | *** | *Leuconostoc* | 2.7 | M | M | 0.52 | ** |
|  | 0.92 | M | M | 0.09 | * |  | 2.7 | M | M | 0.47 | ** |
| *Escherichia-Shigella* | 0.92 | V | V | 0.24 | ** |  | 2.02 | V | V | 0.099 | **** |
|  | 0.61 | M | M | 0.18 | ** |  | 2.02 | V | V | 0.261 | *** |
|  | | | | | | *Bifidobacteriaceae** | 2.68 | M | M | 0.062 | **** |
|  |  |  |  |  |  |  | 2.68 | M | M | 0.02 | **** |
| Stimulus | | | | | |  | 2.02 | V | V | 0.036 | **** |
|  |  |  |  |  |  |  | 2.02 | V | V | 0 | **** |
| Intermediate | | | | | | *Lactococcus* | 2.4 | M | M | 0.171 | *** |
|  |  |  |  |  |  |  | 2.4 | M | M | 0.238 | *** |
| Challenge | | | | | |  | 1.78 | V | V | 0.027 | **** |
|  |  |  |  |  |  |  | 1.78 | V | V | 0.132 | **** |

** Taxonomically unclassified genera were assigned to family rank.*

*** OTHERS refers to all other taxa (other than top 15).*

- 1. **Supplementary Figures**


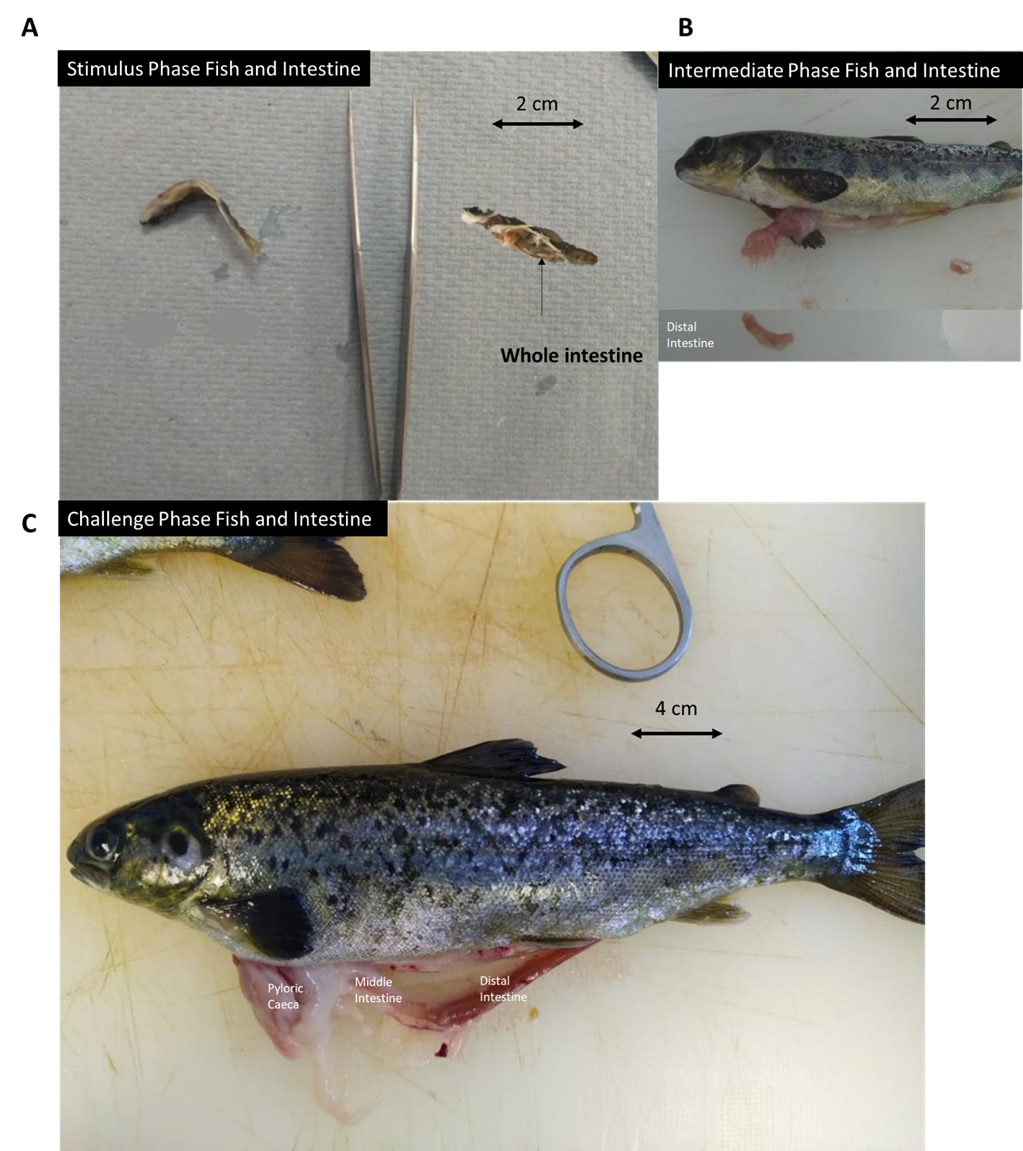


**Fig. S1.** Atlantic salmon fish and intestine at three sampling points at the end of each phase (stimulus, intermediate and challenge); (A) two (B) 14 and (C) 20 weeks post-first feeding. Whole and distal intestines were sampled from the stimulus and challenge phases respectively.


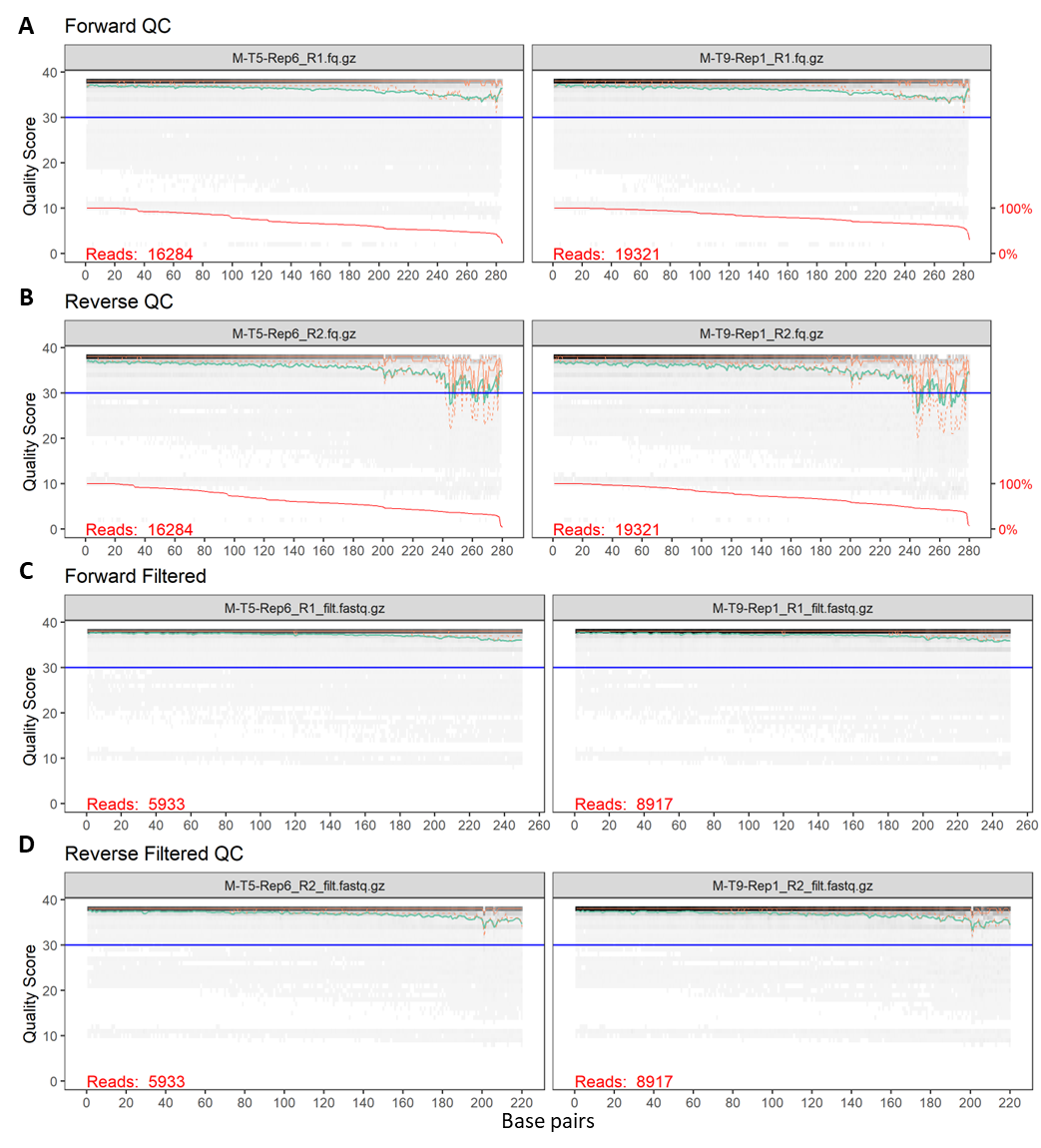


**Fig. S2.** Quality control for forward (F) and reverse (R) reads per sample (two samples used as example) using the DADA2 package before (A and B) and after filtration (C and D). The green line shows the mean quality score at each position, and the orange lines show the quartiles of the quality score distribution. The blue y-intercept line shows a quality score (Q) above 30, the higher the score the better the base call. Filtration at Q > 30 showed up to 250 base pairs (bps) for F reads and 220 bps for R reads thus filtered in. All other samples were checked and the same filtration criteria (Q > 30, 250 bps F, and 220 bps R) were applied. M – marine-based diet fed group (for two weeks at Stim); T – tank number; Rep – replicate number; R1 the sequencer label for a forward read while R2 label for a reverse read. Sequence data output from the sequencer is saved as FASTQ files which are compressed and created with the extension *.fq.gz or *.fastq.gz.


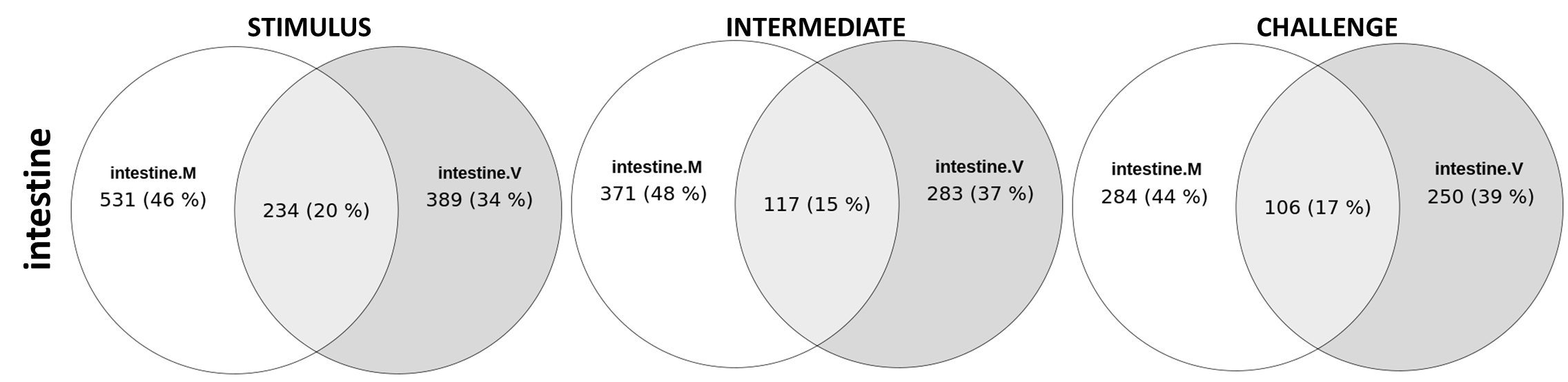


**Fig. S3**. Venn diagram showing unique and shared microbiota ASVs between M and V fish intestine at different phases (stimulus, intermediate, and challenge).


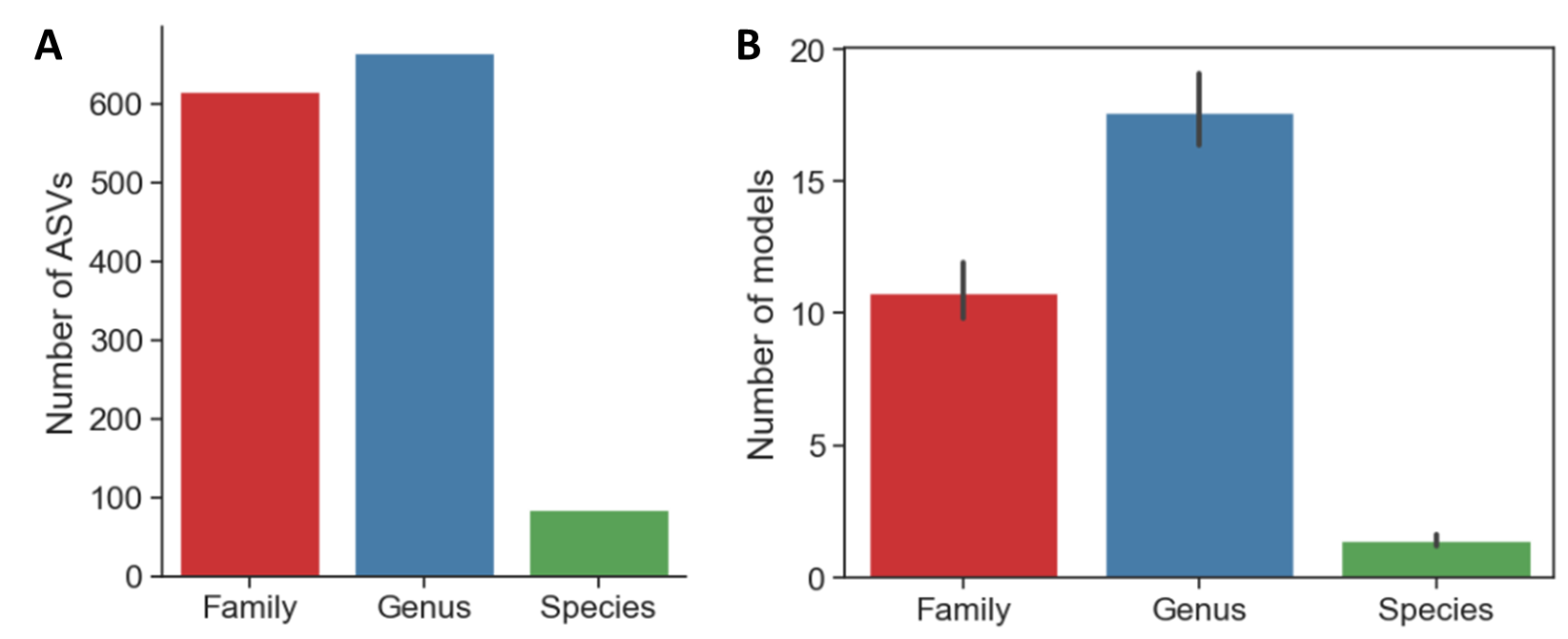


**Fig. S4.** Number of ASVs mapped to genome-scale metabolic models. (A) Number of samples matched to models at different taxonomic levels and (B) the number of models mapped to each sample by taxonomic level.


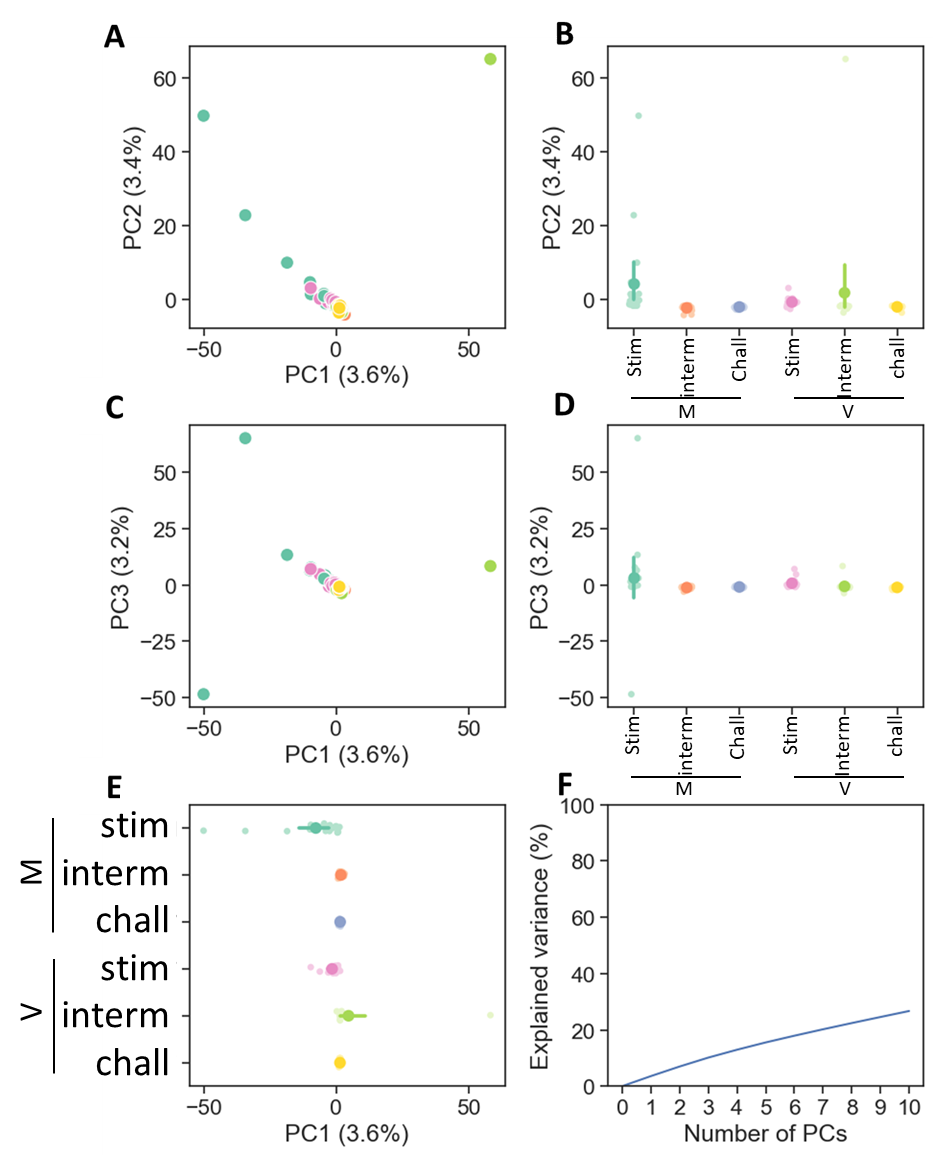


**Fig. S5.** Principal component (PC) analysis on standardized amplicon sequence variants (ASVs). Score plots for; (A) PC1 and PC2; (B) PC2; (C) PC1 and PC3; (D) PC3 and (E) mean scores with 95% confidence intervals for PC1 and (F) percentage of variance explained by PCs. stim:stimulus, interm: intermediate, chall: challenge.


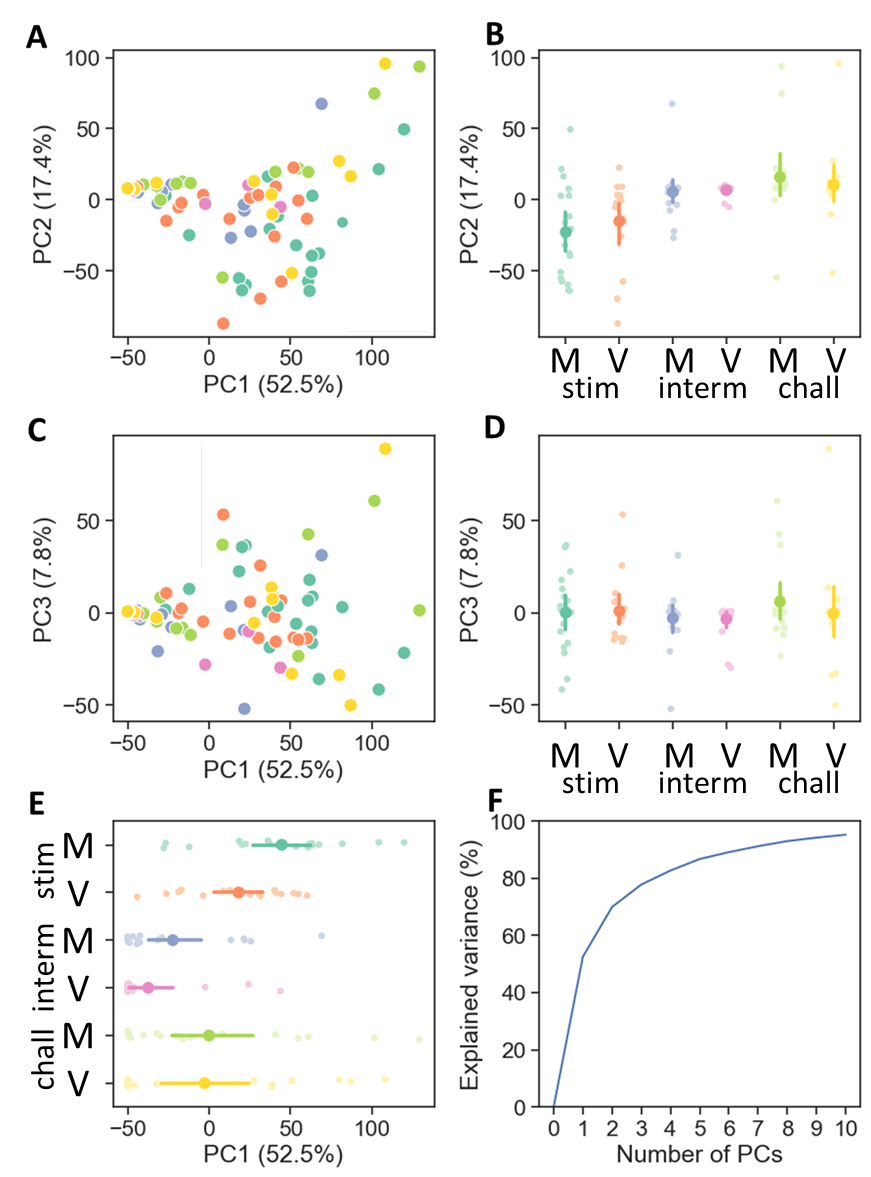


**Fig. S6.** Principal component analysis (PCA) on metabolic reaction abundances (z-scores). Score plots for (A) PC1 and PC2; (B) PC2; (C) PC1 and PC3; (D) PC3 and (E) mean scores with 95% confidence intervals for PC1 and (F) percentage of variance explained by PCs. stim:stimulus, interm: intermediate, chall: challenge.

**List of tables**

**Table 1.** PERMANOVA (global and pairwise) analysis (999 permutations) on Bray-Curtis distances for beta-diversity of microbiota comparisons in Atlantic salmon grouped by fish groups or phase for intestinal samples (n = 18/treatment). For sample type comparisons (intestine n = 105, water or feed n = 3), PERMANOVA analysis was carried out on robust Aitchison distances. SumsOfSqs: sum of squares.

**List of figures**

**Figure 1.** Details of dietary manipulations performed on Atlantic salmon with three sampling points for microbiota samples. The collection gut (n = 108) and water (n = 18) occurred at sampling points and feed (n = 6) at the start of the feeding trial. Experimental groups/dietary regimes are M fish/water and V fish/water for intestinal and water samples.

**Figure 2.** Shannon-Weiner estimate of alpha diversity of the intestinal microbiota of fish fed the experimental diets at different phases (A and B) and across all phases (C). Statistical significance (MWW-tested and BH-corrected) is shown represented by * (p < 0.05), *** (p < 0.001) between different groups and treatments (n = 18/treatment).

**Figure 3.** PCoA of the beta diversity of microbiota in the intestine (Bray-Curtis distances) of M fish (A), V fish (B), and intestine, water, and feed (robust Aitchison distances) (left panel of C). The statistical significance of the permutation test for homogeneity of multivariate dispersions (PERMDISP) shows water is the highest and intestine is the lowest (right panel of C).

**Figure 4.** Relative abundance (as % from 100% total on the y-axis) of the microbiota of the intestine, water, and feed associated with different dietary groups in response to M vs V diets at phylum (A) and genus (B) levels. Individual and grouped (averaged) samples are presented on the left and right panels, respectively.

**Figure 5.** Relative abundance of the top four most abundant phyla in the intestine of salmon fed the experimental diets. The samples are grouped by phases, and the fish dietary group and significant difference (MWW-tested and BH-corrected) is represented by * (p < 0.05) ** (p < 0.01) *** (p < 0.001) **** (p < 0.001).

**Figure 6.** The shared ASVs numbers and relative abundance between the intestine and feed (A) and between intestine and the water (B). ASVs with minimum relative abundance in a sample of 0.05% were included. The M and V fish are labelled across the phases (stimulus, intermediate and challenge).

**Figure 7.** Metabolic reactions analysis using t-tests comparing reaction abundances between (A) M vs V fish at each phase, (B) M fish group and (C) V fish group. The t-statistic for each reaction and the mean across all reactions with a 95% confidence interval for all significantly enriched subsystems.

**List of supplementary tables**

**Table S1.** Formulation, proximate and fatty acid compositions of standard marine diets (MS and MI) and low fishmeal/fish oil vegetable-based diets (VS and VC) used in respective feeding phases.

**Table S2.** Statistical analysis of Shannon-Weiner index of intestine, water and feed comparisons, n denotes to the sample number. p.adj: adjusted p-value. Significant difference (MWW-tested and BH-corrected) is represented by * (p < 0.05) ** (p < 0.01) *** (p < 0.001) **** (p < 0.001). We used the same legend color in figures for different phases.

**Table S3.** Taxonomic composition relative abundances in each of the sample type, phase, and fish group. See taxonomic.composition_devStages in https://github.com/marwa38/NP_devStages_ampliseq to download.

**Table S4.** Top 15 genera (or lowest identified taxonomic rank i.e., family) that showed statistically significant difference (MWW-tested and BH-corrected) between fish groups relative abundances (RA, %). We used the same legend color in figures for different phases. Stars represent corrected statistical significance strength (* p < 0.05, ** p < 0.01, *** p < 0.001, **** p < 0.0001).

**List of supplementary figures**

**Fig. S1.** Atlantic salmon fish and intestine at three sampling points at the end of each phase (stimulus, intermediate and challenge); (A) two (B) 14 and (C) 20 weeks post-first feeding. Whole and distal intestines were sampled from the stimulus and challenge phases respectively.

**Fig. S2.** Quality control for forward (F) and reverse (R) reads per sample (two samples used as example) using the DADA2 package before (A and B) and after filtration (C and D). The green line shows the mean quality score at each position, and the orange lines show the quartiles of the quality score distribution. The blue y-intercept line shows a quality score (Q) above 30, the higher the score the better the base call. Filtration at Q > 30 showed up to 250 base pairs (bps) for F reads and 220 bps for R reads thus filtered in. All other samples were checked and the same filtration criteria (Q > 30, 250 bps F, and 220 bps R) were applied. M – marine-based diet fed group (for two weeks at Stim); T – tank number; Rep – replicate number; R1 the sequencer label for a forward read while R2 label for a reverse read. Sequence data output from the sequencer is saved as FASTQ files which are compressed and created with the extension *.fq.gz or *.fastq.gz.

**Fig. S3.** Venn diagram showing unique and shared microbiota ASVs between M and V fish intestine at different phases (stimulus, intermediate, and challenge).

**Fig. S4.** Number of ASVs mapped to genome-scale metabolic models. (A) Number of samples matched to models at different taxonomic levels and (B) the number of models mapped to each sample by taxonomic level.

**Fig. S5.** Principal component (PC) analysis on standardized amplicon sequence variants (ASVs). Score plots for; (A) PC1 and PC2; (B) PC2; (C) PC1 and PC3; (D) PC3 and (E) mean scores with 95% confidence intervals for PC1 and (F) percentage of variance explained by PCs. stim:stimulus, interm: intermediate, chall: challenge.

**Fig. S6.** Principal component analysis (PCA) on metabolic reaction abundances (z-scores). Score plots for (A) PC1 and PC2; (B) PC2; (C) PC1 and PC3; (D) PC3 and (E) mean scores with 95% confidence intervals for PC1 and (F) percentage of variance explained by PCs. stim:stimulus, interm: intermediate, chall: challenge.
